# Accelerating Adaptation of Natural Resource Management to Address Climate Change

**DOI:** 10.1111/j.1523-1739.2012.01954.x

**Published:** 2012-10-30

**Authors:** Molly S Cross, Patrick D McCarthy, Gregg Garfin, David Gori, Carolyn AF Enquist

**Affiliations:** * Wildlife Conservation Society301 N. Willson Avenue, Bozeman, MT, 59715, U.S.A.; † The Nature Conservancy212 E. Marcy Street, Suite 200, Santa Fe, NM, 87501, U.S.A.; ‡ University of Arizona845 N. Park Avenue, Ste. 532, Tucson, AZ, 85721, U.S.A.; § USA National Phenology Network, National Coordinating Office1955 E. Sixth Street, Tucson, AZ, 85721, U.S.A.

**Keywords:** climate change, community of practice, conservation planning, learning networks, natural resources, Cambio climático, comunidad de práctica, planificación de la conservación, recursos naturales, redes de aprendizaje

## Abstract

**Abstract:**

Natural resource managers are seeking tools to help them address current and future effects of climate change. We present a model for collaborative planning aimed at identifying ways to adapt management actions to address the effects of climate change in landscapes that cross public and private jurisdictional boundaries. The Southwest Climate Change Initiative (SWCCI) piloted the Adaptation for Conservation Targets (ACT) planning approach at workshops in 4 southwestern U.S. landscapes. This planning approach successfully increased participants’ self-reported capacity to address climate change by providing them with a better understanding of potential effects and guiding the identification of solutions. The workshops fostered cross-jurisdictional and multidisciplinary dialogue on climate change through active participation of scientists and managers in assessing climate change effects, discussing the implications of those effects for determining management goals and activities, and cultivating opportunities for regional coordination on adaptation of management plans. Facilitated application of the ACT framework advanced group discussions beyond assessing effects to devising options to mitigate the effects of climate change on specific species, ecological functions, and ecosystems. Participants addressed uncertainty about future conditions by considering more than one climate-change scenario. They outlined opportunities and identified next steps for implementing several actions, and local partnerships have begun implementing actions and conducting additional planning. Continued investment in adaptation of management plans and actions to address the effects of climate change in the southwestern United States and extension of the approaches used in this project to additional landscapes are needed if biological diversity and ecosystem services are to be maintained in a rapidly changing world.

Acelerando la Adaptación del Manejo de Recursos Naturales para Atender el Cambio Climático

**Resumen:**

Los manejadores de recursos naturales están buscando herramientas para ayudarles a atender los efectos actuales y futuros del cambio climático. Presentamos un modelo para la planificación colaborativa enfocada a identificar formas para adaptar las acciones de manejo para atender los efectos del cambio climático en paisajes que cruzan límites jurisdiccionales públicos y privados. La Iniciativa Sudoccidental de Cambio Climático (ISCC) puso a prueba el método de planificación de Adaptación para Metas de Conservación (AMC) en talleres en cuatro paisajes del suroeste de E. U. A. Este método de planificación incrementó exitosamente la capacidad de los participantes para atender el cambio climático al proporcionarles un mejor entendimiento de los efectos potenciales y guiar la identificación de soluciones. Los talleres promovieron el diálogo trans-jurisdiccional y multidisciplinario sobre cambio climático mediante la participación activa de científicos y manejadores en la evaluación de efectos del cambio climático, la discusión de implicaciones de esos efectos para determinar las metas y actividades de manejo y desarrollar oportunidades para la coordinación regional de la adaptación de planes de manejo. La aplicación simplificada del marco AMC llevó las discusiones de grupo más allá de la evaluación de los efectos a la concepción de opciones para mitigar los efectos del cambio climático sobres determinadas especies, funciones ecológicas y ecosistemas. Los participantes abordaron la incertidumbre de las condiciones futuras al considerar más de un escenario de cambio climático. Delinearon oportunidades e identificaron los siguientes pasos para la implementación de varias acciones, y asociaciones locales han comenzado a implementar acciones y realizar planificación adicional. Se requiere inversión continua en la adaptación de planes y acciones de manejo para atender los efectos del cambio climático en el suroeste de Estados Unidos y la extensión de los métodos utilizados en este proyecto en paisajes adicionales si se quiere mantener la diversidad biológica y los servicios de los ecosistemas en un mundo que cambia rápidamente.

## Introduction

Global average annual temperature has increased by 1.3 °C over the last 50 years and is expected to increase by another 2–5 °C over the next century (Solomon et al. 2007). Ecological responses to warming and related climate changes suggest that natural resource managers may need to reconsider conventional goals, plans, and practices (National Research Council 2010). Recognition of the need for climate-change adaptation (i.e., “adjustment in natural or human systems to a new or changing environment that exploits beneficial opportunities or moderates negative effects” [National Research Council 2010]) is spreading, but capacity for taking action is lacking. Resource managers are often stymied by complexity and uncertainty in climate-change scenarios, lack of knowledge about local effects, and the absence of readily apparent ways to respond (Lawler et al. 2010). Recent climate-adaptation planning efforts illustrate that constructive dialogue between scientists and managers that focuses on local interpretation of climate-change projections and ecological responses can help overcome these barriers and produce pragmatic and evidence-based strategies for climate-change adaptation (e.g., Halofsky et al. 2011). Practical models for facilitating this type of collaborative adaptation planning can help guide managers as they search for ways to integrate climate change into management.

## Model for Collaborative Adaptation Planning

**Figure 1:**
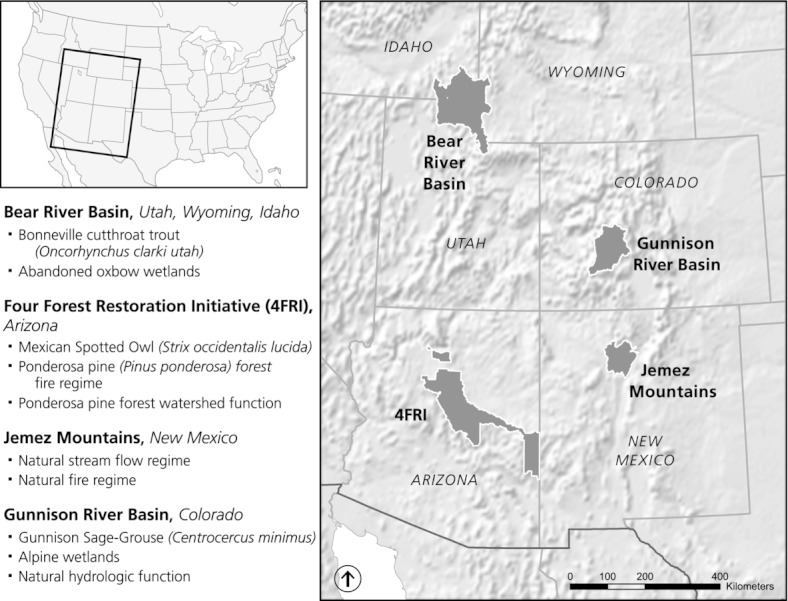
Focal landscapes and conservation features addressed during Southwest Climate Change Initiative climate-change adaptation planning workshops (figure created by Steve Bassett, The Nature Conservancy).

In southwestern United States—the semiarid region that includes the states of Arizona, Colorado, New Mexico, and Utah (Fig. 1)—conservation practitioners have called for knowledge and tools that will help them sustain species and ecosystems as climate changes (McCarthy et al. 2008). Many climate models project that by the middle of the 21^st^ century the region will be dominated by conditions similar to those of the 1930s drought, including exceedingly low rainfall, high temperatures, high winds, and dust storms (Seager et al. 2007). To build capacity among natural resource managers for understanding and responding to such changes, The Nature Conservancy (TNC); Climate Assessment for the Southwest (University of Arizona); Wildlife Conservation Society; Western Water Assessment (University of Colorado-Boulder); University of Washington; U.S. Forest Service; and National Center for Atmospheric Research joined together to create the Southwest Climate Change Initiative (SWCCI) (McCarthy 2012). Modeled after the U.S. Fire Learning Network (Goldstein & Butler 2010), the primary goal of the SWCCI is to cultivate expertise in climate-change science and adaptation planning by linking local practitioners and managers to a regional community of practice.

As one approach to meeting this goal, the SWCCI piloted the Adaptation for Conservation Targets (ACT) framework, a new tool for facilitating collaborative climate-change adaptation planning and action for natural resource management (Cross et al. 2012). The ACT framework complements other approaches to adaptation planning by offering a simple stepwise process for identifying adaptation actions for particular conservation features (e.g., species, ecosystems, and ecological functions) that encourages participation of multiple public and private jurisdictions and uses scenario planning to address uncertainties related to projecting future climate and ecological conditions. The SWCCI implemented the ACT framework during a series of workshops with resource professionals and scientists at 4 landscapes across southwestern United States. Each workshop aimed to facilitate cross-jurisdictional, multidisciplinary dialogue on the consequences of climate change for selected features, move participants beyond simply assessing effects to identifying practical strategies for reducing adverse effects on those features, and accelerate the implementation of priority adaptation strategies. The SWCCI-led ACT framework workshops are a model for initiating targeted, collaborative adaptation planning among diverse partners.

## Workshops

Between April 2009 and May 2010, the SWCCI conducted one adaptation-planning workshop in each of 4 pilot landscapes. In one-on-one and large-group consultations with scientists and managers, we selected one pilot landscape in each state on the basis of its conservation significance as identified in ecoregional assessments (Marshall et al. 2006) and state and federal agency conservation plans; climate-change exposure (i.e., changes in mean annual temperatures from 1951 to 2006 [Robles & Enquist 2010] and mean annual water deficit between 1970 and 2006 [Enquist et al. 2008]); strength of local conservation partnerships; and availability of place-specific scientific information about climate change and its ecological effects. Although most of the natural landscapes of the Southwest have been or will be affected by climate change, we chose 4 landscapes that the consulting scientists and managers agreed were at high risk of species extirpation, loss of ecosystem services, or other undesirable changes: Jemez Mountains, upper Gunnison River basin, Bear River basin, and 4 National Forests and surrounding lands that comprise the U.S. Forest Service's Four Forest Restoration Initiative (Fig. 1). Before each workshop, we solicited participant input via surveys and interviews to select 2 or 3 features to serve as the focus of adaptation-planning exercises (Fig. 1).

**Figure 2:**
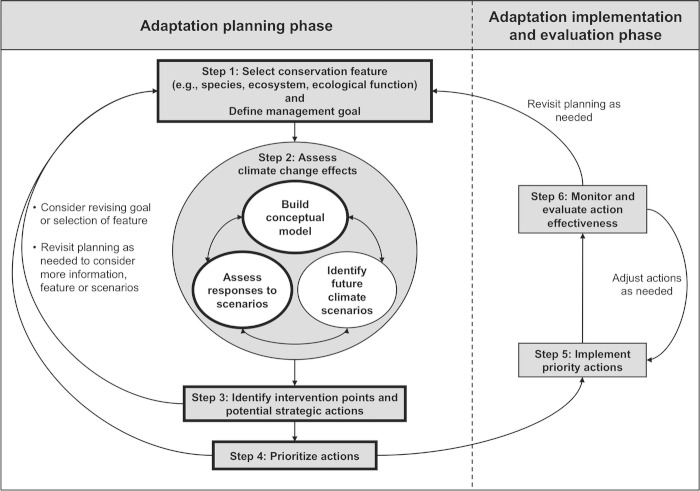
Steps in the Adaptation for Conservation Targets (ACT) approach to climate-change adaptation planning and action (reprinted with kind permission from Springer Science+Business Media: Environmental Management, The Adaptation for Conservation Targets [ACT] framework: a tool for incorporating climate change into natural resource management. Volume 50, 2012, p. 343, Cross et al., Fig. 2). Facilitators led participants through the planning phase steps outlined in bold during the Southwest Climate Change Initiative workshops.

**Figure 3:**
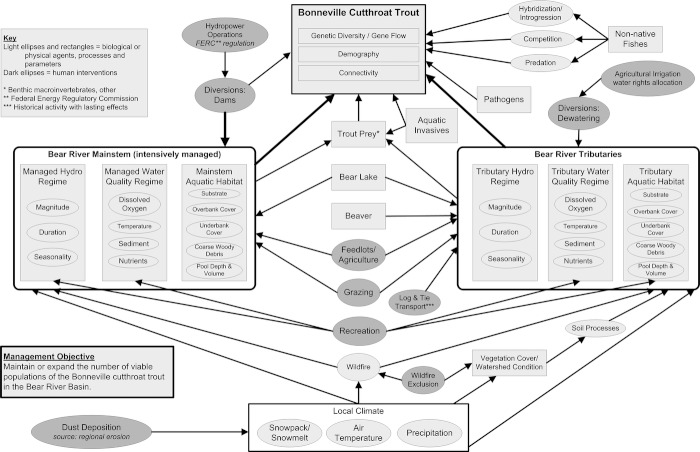
Conceptual model illustrating key climatic, physical, ecological, and socioeconomic drivers that affect Bonneville cutthroat trout in the Bear River basin of Utah, Wyoming, and Idaho.

Each workshop followed a similar format that included introductory presentations, small-group adaptation-planning exercises, and full-group discussions of challenges, opportunities, and next steps for action (see Supporting Information for additional workshop methods). During small-group breakout sessions, facilitators led participants through the planning steps of the ACT framework (Fig. 2 & Supporting Information). In the first step, participants specified a management goal for the feature being addressed. In the second step, they built a conceptual model that illustrated the climatic, physical, ecological, and socioeconomic drivers that affect that feature (Fig. 3). Next, participants assessed the effects of 2 plausible future climate scenarios that we developed in collaboration with climate and hydrology experts from the region before the workshops (Supporting Information). After identifying potential adaptation actions for each scenario, participants highlighted several high-priority actions on the basis of relative feasibility, effectiveness, cost, and their applicability under both scenarios. Finally, workshop facilitators and participants engaged in a plenary discussion about how to collaboratively implement high-priority adaptation strategies. Summary reports for each workshop documented discussions and provided detailed adaptation plans for each conservation feature, including conceptual models (Fig. 3), expert assessments of the effects of climate change for 2 future climate scenarios, and proposed strategic actions (Table 1) (Enquist et al. 2009; Degiorgio et al. 2010; Neely et al. 2010; Smith et al. 2011).

**Table 1:** Example climate-adaptation actions recommended for achieving management goals in light of actual or hypothesized climate change effects under 2 climate scenarios^a^ considered at each Southwest Climate Change Initiative workshop

*Landscape*	*Conservation feature*	*Management goal*	*Actual or hypothesized climate-change effects*	*Strategic adaptation actions*
Jemez Mountains, New Mexico	natural stream flow regime	maintain sufficient water in the system to support aquatic species and riparian vegetation.	reduced snowpack and greater variability in precipitation; reduced stream base flows	restore beaver to streams build artificial structures to increase floodplain aquifer recharge
				apply forest thinning treatments that maximize snowpack retention and provide optimal shade to minimize sublimation and evaporation losses
Gunnison River basin, Colorado	Gunnison Sage-Grouse (*Centrocercus minimus*)	increase and maintain the Gunnison population of Sage-Grouse at >3500 individuals and the Crawford population at >200 individuals.	loss of nesting habitat due to increased fire frequency, cheatgrass invasion, and sagebrush dieback; decreased habitat quality due to a decline in forbs and perennial grasses; reduced recruitment	improve or restore nesting and wintering habitats improve or reestablish leeward mountain shrub habitats (e.g., snowberry, serviceberry) via fencing and planting
				maintain and expand perennial grass and forb cover by planting and fencing; abate or prevent cheatgrass encroachment by spraying
Four Forests Restoration Initiative area, Arizona	Ponderosa pine (*Pinus ponderosa*) forest watershed function	maintain or improve watershed function in systems dominated by ponderosa pine by maintaining and improving water quality, quantity, and timing of flow for surface and ground water; soil productivity; and recharge-to-runoff ratio.	increased temperature leads to increased potential evapotranspiration and decreased recharge; increased moisture stress for plants and lower base flows in rivers and streams that affect aquatic species	apply forest-restoration treatments (e.g., thinning, controlled burns) to reduce fire risk and drought-induced tree mortality, increase herbaceous ground cover, and enhance infiltration, soil moisture and recharge
				plan for 6-year (on average) fire rotation to maintain water yield benefits
Bear River basin, Utah/ Wyoming/Idaho	Bonneville cutthroat trout (*Oncorhynchus clarki utah*)	maintain or expand the number of viable populations of Bonneville cutthroat trout in the Bear River Basin by maintaining or restoring Bonneville cutthroat trout habitat, ecology and life history.	higher air temperatures increase evapotranspiration, decrease summer base flow, and raise summer water temperature, resulting in an expansion of uninhabitable reaches	restore connectivity between river mainstem and tributaries by rewatering streams to facilitate trout dispersal protect habitat in reaches that provide thermal refugia
				lower the depth of water outflow from hydropower and irrigation reservoirs to reduce downstream water temperature

a Future climate scenarios for each workshop are detailed in Supporting Information. Although many of the actions identified at each workshop were considered applicable under both climate scenarios (as is the case with the examples provided here), there were examples where different, additional, or modified actions were identified for the second scenario. Complete lists of adaptation actions identified for each conservation feature can be found in landscape-specific workshop reports at http://bit.ly/jnerFG.

Roughly 45–60 participants attended each workshop, and there were 15–20 participants in each breakout group. In total, 190 natural resource managers, scientists, and conservation practitioners from 44 local, state, tribal, and federal agencies and organizations attended the 4 workshops (Supporting Information). Participants’ baseline knowledge of climate change varied, and they articulated a number of factors that inhibited their ability to take action on addressing the effects of climate change. In preworkshop surveys and facilitated discussions at the start of each workshop, participants named several barriers to adaptation including uncertainty about future climate and ecological conditions, lack of understanding of how to apply existing climate science to decision making, and lack of support from agency leaders and the public for taking local action on climate change (Supporting Information). To maximize opportunities for participants to interact, only one-quarter of each workshop was spent on one-way presentations of information, with the remaining 3-quarters dedicated to small-group breakout sessions and large-group discussions (Supporting Information).

To measure participants’ perceptions of the presentations, breakout sessions, plenary discussions, and the workshop as a whole, we conducted written surveys at the end of the workshops in Colorado and Arizona (Supporting Information). We conducted written surveys before the last 2 workshops in Arizona and Utah (Supporting Information) to assess invited participants’ interests and needs related to climate change and climate-change adaptation. We used results from both types of surveys to design preworkshop webinars on climate change (Supporting Information) and to refine the format of subsequent workshops. Because the workshops were spread out over 13 months, we were able to apply lessons learned in the earliest workshops to subsequent workshops.

Exit surveys indicated that participants thought the ACT workshop improved their understanding of climate change and its local effects. For example, 33 of 35 respondents (94%) to the Gunnison River basin exit survey said they “mostly” or “absolutely” gained a better understanding of climate change on the Gunnison Basin (Supporting Information). Thirty of 34 respondents (88%) thought the framework presented was “mostly” or “absolutely” useful for developing climate adaptation strategies (Supporting Information). Twenty-nine out of 33 respondents (88%) said they “mostly” or “absolutely” had a better understanding of the resources that are available and how to incorporate climate-adaptation strategies into their conservation work (Supporting Information). Although at least 50% of respondents indicated that each of the Gunnison River basin workshop activities “mostly” or “absolutely” provided valuable information that enhanced their understanding of climate adaptation issues, some activities were rated more favorably than others (Supporting Information). Exit surveys from the Four Forest Restoration Initiative workshop yielded similar feedback (Supporting Information).

Although workshop format and content was generally consistent across all 4 workshops, we made many adjustments in later workshops to address time constraints. For example, for the first 2 workshops we had participants create conceptual models during breakout sessions. This took a great deal of workshop time and resulted in less time for later planning steps. To more efficiently use time during subsequent workshops, we asked participants to refine prepared draft models. We also reduced the amount of time spent on introductory presentations during the last 2 workshops by offering a preworkshop webinar on basic climate-change science. For the Colorado, Arizona, and Utah workshops, we incorporated a few elements of TNC's Conservation Action Planning guidance on climate change (TNC 2009) into the ACT approach. These slight modifications included integrating terminology from the Conservation Action Planning method (e.g., “hypotheses of change” and “strategic adaptation actions”) and prompting participants to discuss ways in which human responses to climate change may affect the focal feature. Finally, we changed our approach to developing climate scenarios over the course of the 4 workshops (described in Supporting Information). Taken together, we believe these refinements streamlined the planning process and made the inevitably time-limited workshops more productive.

## Facilitating Multidisciplinary Dialogue across Jurisdictions

Active engagement of a diversity of scientists and managers in a collaborative process allowed us to reach our goal of fostering cross-jurisdictional and multidisciplinary dialogue on climate change in each of the 4 workshop sites. The workshops provided a structured forum for developing a shared understanding of the effects of climate change, identifying options for adjusting management goals and strategies in light of those effects, and identifying opportunities for interagency and regional coordination. These elements of stakeholder involvement are critical to motivating climate-change adaptation action (Kloprogge & van der Sluijs 2006).

The coproduction of knowledge by scientists and resource managers (sensu Lemos & Morehouse 2005) is a cornerstone of the ACT framework. For example, managers and scientists articulated their collective understanding of ecological, physical, and socioeconomic drivers under current and future climates when refining conceptual models and assessing the potential effects of climate change. Moreover, by capitalizing on available local knowledge and expertise about ecological systems, rather than requiring new or extensive modeling, the ACT framework cultivated practical and efficient problem solving. Another demonstrated benefit of this approach was that it helped establish and strengthen local scientist-manager partnerships that could be used to identify and address priority information needs.

## Moving beyond Assessment of Climate-Change Effects

Facilitated application of the ACT planning steps helped participants move beyond assessing the effects of climate change to engaging in solution-oriented discussions about designing new and retrofitting existing conservation actions for climate change. As one U.S. Fish and Wildlife Service manager expressed during closing remarks of one workshop:

What I valued most about this workshop was its step-by-step process that takes you from being overwhelmed to identifying targeted actions that will address the problem. I am the point person in my office to attend these climate change workshops, which are mostly just depressing. This one was different because there was a focus on the huge variety of things that we can do (Degiorgio et al. 2010).

This dialog produced concrete adaptation actions and stimulated discussion about the long-term feasibility of current management goals.

### Identifying Concrete Actions

The ACT approach focuses on a finite set of conservation features to make adaptation discussions as concrete as possible while encouraging participants to describe adaptation actions in as much detail as time allows. The SWCCI workshops succeeded in producing some relatively specific adaptation actions, such as “improve or reestablish leeward mountain shrub habitats via fencing and planting” for Gunnison Sage-Grouse (*Centrocercus minimus)* (Neely et al. 2010). Other actions, however, were more broadly stated, such as “increase connectivity to allow range shifts” for Bonneville cutthroat trout (*Oncorhynchus clarki utah)* (Degiorgio et al. 2010). Time constraints limited the ability of participants to explore all adaptation options in detail. Although the ACT steps promote targeted thinking about adaptation options, more time is needed to fully develop the adaptation options identified during a 2-day workshop.

Nonetheless, participants considered many different types of adaptation strategies, including: land and water protection; land, water, and species management; and regulatory and policy changes (Enquist et al. 2009; Degiorgio et al. 2010; Neely et al. 2010; Smith et al. 2011). These actions included practices that are familiar to managers (e.g., forest thinning and controlled burns), although participants often recognized that the priority, pace, and extent of such ongoing activities would likely need to be adjusted to address climate-driven ecological changes. Participants also produced unconventional and even controversial ideas such as lowering the depth of water outflow from a hydropower and irrigation reservoir to reduce downstream water temperatures in the Bear River (Table 1) and assisting the migration of pine species adapted to warmer and drier conditions in southern Arizona and northern Mexico into areas that currently support only ponderosa pine (*Pinus ponderosa*) (Smith et al. 2011).

The ACT framework can be applied sequentially to evaluate additional features and build more comprehensive climate-adaptation plans. Information developed during initial ACT framework applications can be used to make the process more efficient during subsequent uses. For example, several organizations are building on the information generated during the initial Jemez Mountains workshop to develop an adaptation plan for the Jemez Mountains salamander (*Plethodon neomexicanus*). The Gunnison Climate Working Group, established by local natural resource managers after the initial SWCCI workshop in the Gunnison Basin, plans to use the ACT framework to identify climate-adaptation strategies for many species and ecosystems that were not considered initially.

### Reconsidering Management Goals

The ACT framework prompts users to consider revising current management goals after identifying potential strategic actions (Fig. 2). In practice, workshop participants questioned the feasibility of current management goals at earlier points in the process such as when they articulated current management goals and as they summarized the likely effects of the 2 climate scenarios on the conservation feature. Users of the ACT framework might therefore consider adjusting the process to prompt a discussion about the feasibility of management goals during those earlier steps.

The process of framing, and then reframing, management goals often occurred in an iterative manner. For example, participants at the Gunnison River basin workshop acknowledged at the start of the breakout sessions that it might not be possible to maintain the current spatial extent of alpine wetlands in the study area. Later in the process, when discussing strategic adaptation options, they tentatively revised the management goal to maintaining at least 75% of current wetlands (Neely et al. 2010). After further discussion, participants recognized that even this modified goal might not be attainable and reported the need to continue reevaluating that goal. Similarly, participants at the Four Forest Restoration Initiative workshop considered whether the recovery program goal for the federally listed Mexican Spotted Owl (*Strix occidentalis lucida*) would be feasible under future climate scenarios at several points during their breakout session. They ultimately revised the goal to accommodate hypothesized changes in habitat distribution (Smith et al. 2011).

Despite the fact that participants identified major climate-related risks for several of the conservation features and discussed the need to revisit current management goals, none of the breakout groups recommended abandoning the conservation of a feature altogether. More work is needed to understand whether participants think such dramatic revision of goals is unnecessary or whether it reflects a “general cautiousness” about embracing unavoidable change, as suggested by Poiani et al. (2011).

### Coping with Uncertainty

The ACT framework uses scenario planning to help managers address uncertainties about the pace and magnitude of climate change and its ecological effects (Mahmoud et al. 2009). It is especially useful in identifying adaptation actions that are likely to be effective under multiple climate scenarios and are therefore more robust to uncertainty in future conditions. Several researchers recommend focusing on these so-called no-regret actions (e.g., Willows & Connell 2003) and prioritizing them for near-term implementation.

Before each workshop, we worked closely with climatologists, hydrologists, and ecologists to develop scenarios that would be useful to understanding the effects of climate change on the selected features (Supporting Information). The inclusion of more than one scenario allowed for exploration of how climate change effects, and therefore adaptation options, might differ across scenarios. Despite some differences across scenarios, many of the adaptation actions identified by workshop participants were recommended under both climate-change scenarios (e.g., those presented in Table 1 and others detailed in Enquist et al. 2009, Degiorgio et al. 2010, Neely et al. 2010, and Smith et al. 2011). However, in some cases, participants identified the need for different, additional, or modified actions under the second climate scenario. For example, participants in the Jemez Mountains workshop indicated that more extreme contingency plans might need to be considered under the even hotter and drier “mega-drought” scenario (Supporting Information), such as pumping water from the lower end of a stream segment to the top to augment flow for focal species (Enquist et al. 2009).

There are several possible reasons that workshop participants rarely recommended different adaptation actions for the 2 climate-change scenarios presented. First, there was often limited time available to discuss the ecological effects of the second scenario in detail. Second, the participants’ discussion of climate-change effects frequently emphasized qualitative changes. This emphasis could result in missed opportunities to identify and respond to quantitative ecological thresholds, such as drought-induced tree mortality. This may have been the case for landscapes for which the second scenario was a more extreme version of the first (i.e., changes were in the same direction but were of greater magnitude) (e.g., see Jemez Mountains scenarios in Supporting Information). Even when the climate scenarios differed in the direction or seasonality of precipitation changes (e.g., see Bear River scenarios in Supporting Information), the hydrological consequences of those scenarios were sometimes similar and led to comparable recommended actions for aquatic species and ecosystems.

Although the workshop outputs suggest there may be many no-regret management actions for these features, additional work is needed to more thoroughly assess whether those actions are robust to uncertainty when they are applied under a full range of possible future climates. Such work could provide greater clarity as to the sensitivity of adaptation strategies to differences among climate-change projections. For example, robust strategies may be more readily identified when projected climatic changes are insufficiently different among scenarios, when the ecological effects of those climate changes are insufficiently different across scenarios, when the focal system or species is insensitive to differences across projected climate or ecological scenarios, or when the action itself is insensitive to the range of projected climatic or ecological changes across scenarios. It may also be necessary to consider more than just 2 alternative scenarios to capture the full range of plausible trajectories.

## Accelerating Adaptation Action

The SWCCI workshops included several elements designed to accelerate the implementation of identified adaptation actions. The 4 pilot landscapes were selected, in part, because of the strength of local conservation partnerships. We expected this would increase the likelihood that participants, including SWCCI member organizations, would follow through on workshop recommendations. We also expected that managers who had participated in the workshops would have a stake in the recommendations and therefore would be more likely to implement them. At each workshop, we tried to guide participants to identify specific opportunities, costs, necessary partnerships, and next steps for implementation of high-priority actions (Enquist et al. 2009; Degiorgio et al. 2010; Neely et al. 2010; Smith et al. 2011). Each workshop closed with a plenary discussion focused on next steps. Efforts are now underway in all 4 landscapes to expand on the initial SWCCI workshops to conduct further planning and to move from planning to implementation of on-the-ground adaptation strategies (McCarthy 2012).

The SWCCI workshops and ACT planning steps provided a valuable launching point for multijurisdictional climate-change adaptation planning and action in the pilot landscapes. However, for adaptation planning to be both rigorous and comprehensive, more analyses and dialogue is needed than can be completed in a single 2-day workshop. Success will require additional engagement of local scientists and managers toward refining understanding of climate-change effects across a broad range of plausible climate scenarios; evaluating and modifying management goals; refining and setting priorities among adaptation strategies; considering additional conservation features; and constituency building and fund raising for implementation.

We recommend the ACT framework be applied in the context of sustained science-management partnerships, such as the regional SWCCI and associated local working groups, that bring together diverse organizations and disciplines. Continued investment in climate-adaptation planning and action and extension of the approaches used in this project to additional landscapes are needed to sustain biological diversity and ecosystem services in a rapidly changing world.

## References

[b1] Cross MS (2012). The Adaptation for Conservation Targets (ACT) framework: a tool for incorporating climate change into natural resource management. Environmental Management.

[b2] Degiorgio J, McCarthy P, Cross MS, Garfin G, Gori D, Tuhy J (2010). Bear River climate change adaptation workshop summary.

[b3] Enquist C, Bradley A, Cross M, Garfin G, Gori D, McCarthy P, Oertel R (2009). Jemez Mountains climate change adaptation workshop: process, outcomes and next steps.

[b4] Enquist CAF, Girvetz E, Gori D (2008). A climate change vulnerability assessment for biodiversity in New Mexico, part 2: conservation implications of emerging moisture stress due to recent climate changes in the southwestern United States.

[b5] Goldstein BE, Butler WH (2010). Expanding the scope and impact of collaborative planning. Journal of the American Planning Association.

[b6] Halofsky JE, Peterson DL, O'Halloran KA, Hawkins-Hoffman C (2011). Adapting to climate change at Olympic National Forest and Olympic National Park. General technical report PNW-GTR-844. U.S.

[b7] Kloprogge P, van der Sluijs JP (2006). The inclusion of stakeholder knowledge and perspectives in integrated assessment of climate change. Climatic Change.

[b8] Lawler JJ (2010). Resource management in a changing and uncertain climate. Frontiers in Ecology and the Environment.

[b9] Lemos MC, Morehouse BJ (2005). The co-production of science and policy in integrated climate assessments. Global Environmental Change.

[b10] Mahmoud M (2009). A formal framework for scenario development in support of environmental decision-making. Environmental Modelling & Software.

[b11] Marshall R, List M, Enquist C (2006). Ecoregion-based conservation assessments of the southwestern United States and northwestern Mexico: a geodatabase for six ecoregions, including the Apache Highlands, Arizona-New Mexico Mountains, Colorado Plateau, Mojave Desert, Sonoran Desert, and Southern Rocky Mountains.

[b12] McCarthy PD (2012). Climate change adaptation for people and nature: a case study from the U.S. Southwest. Advances in Climate Change Research.

[b13] McCarthy PD, Enquist CAF, Garfin G (2008). Mitigating climate change in the American Southwest. Eos, Transactions American Geophysical Union.

[b14] National Research Council (2010). America's climate choices: adapting to the impacts of climate change.

[b15] Neely B, McCarthy P, Cross M, Enquist C, Garfin G, Gori D, Hayward G, Schulz T (2010). Climate change adaptation workshop for natural resource managers in the Gunnison Basin: summary.

[b21] Poiani KA, Goldman RL, Hobson J, Hoekstra J, Nelson K (2011). Redesigning biodiversity conservation projects for climate change: examples from the field. Biological Conservation.

[b16] Robles MD, Enquist CAF (2010). Managing changing landscapes in the southwestern United States.

[b22] Seager R, Ting MF, Held I, Kushnir Y, Lu J, Vecchi G, Huang H, Harnik N, Leetma A, Lau N, Li C, Velez J, Naik N (2007). Model projections of an imminent transition to a more arid climate in southwestern North America. Science.

[b17] Smith E, Cross MS, Garfin G, McCarthy P, Gori D, Robles M, Enquist C (2011). The Four Forest Restoration Initiative: implementing a climate change adaptation framework for natural resource management and planning.

[b18] Solomon S, Qin D, Manning M, Chen Z, Marquis M, Averyt KB, Tignor M, Miller HL (2007). Climate change 2007: the physical science basis. Contribution of Working Group I to the fourth assessment report of the Intergovernmental Panel on Climate Change.

[b19] TNC (The Nature Conservancy) (2009). Conservation action planning guidelines for developing strategies in the face of climate change.

[b20] Willows RI, Connell RK (2003). Climate adaptation: risk, uncertainty and decision-making. United Kingdom Climate Impacts Programme technical report.

