# Supplementary material for: Accelerating Adaptation of Natural Resource Management to Address Climate Change
Source: Conserv Biol. 2012 Oct 30;27(1):4–13. doi: 10.1111/j.1523-1739.2012.01954.x (PMC3562478; doi:10.1111/j.1523-1739.2012.01954.x)
Supplement: Supplementary file 1 [file cobi0027-0004-SD1.doc]

**Supporting Information Appendix S1.**

**ADDITIONAL INFORMATION ON WORKSHOP METHODS**

**Workshop organization**

Each SWCCI workshop was organized by a planning team, a science team, and an operations and logistics team. The lead workshop organizer was from the local Nature Conservancy (TNC) state chapter, and the planning and science teams included staff from several SWCCI founding organizations (TNC, Climate Assessment for the Southwest, and Wildlife Conservation Society), and a few (usually 2-5) representatives from key local organizations. The planning teams developed the workshop goals, objectives, and agenda; recruited expert speakers; identified prospective workshop participants; prepared materials to be distributed to participants before and during the workshop; developed pre-workshop and exit surveys; and organized a pre-workshop webinar on fundamentals of climate change science. The science teams determined the landscape boundaries to be considered at each workshop, selected focal conservation features (e.g., species, ecosystems or ecological functions) on the basis of input from invited participants, prepared draft graphical conceptual models for each conservation feature, and worked with regional climate experts to develop and document climate scenarios. Staff from the local Nature Conservancy state chapter organized workshop logistics such as securing a venue; sending invitations; distributing materials to participants ahead of and during the workshop; gathering workshop supplies such as projectors, flip charts, and white boards; and taking notes during workshop discussions. Members of the planning and science team facilitated workshop discussions and breakout groups. Southwest Climate Change Initiative core team members from TNC, Climate Assessment for the Southwest, and Wildlife Conservation Society helped plan and facilitate all four workshops. The lead workshop organizer worked with facilitators and the planning and science teams to summarize workshop discussions and results in detailed reports that were subsequently distributed to participants and made freely available at <http://bit.ly/jnerFG>.

**Workshop dates**

All workshops were held within a 13-month period: the Jemez Mountains, New Mexico, workshop was held in April 2009; the Gunnison River basin, Colorado, workshop was held in December 2009; the Four Forest Restoration Initiative, Arizona, workshop was held in April 2010; and the Bear River basin, Utah, Wyoming and Idaho, workshop was held in May 2010.

**Workshop participants**

In total, 190 natural resource managers, scientists and conservation practitioners from 44 local, state, tribal and federal agencies and organizations participated in the four workshops (Table S1). Workshop organizers familiar with decision-makers and experts in each landscape were responsible for identifying participants for the workshops. Scientists and managers were selected based on the relevance of their expertise to the conservation features under consideration, involvement of their agencies and organizations in informing or undertaking natural resource management actions, their ability to influence and lead decision-making within their organizations or agencies, and/or their interest in addressing climate change in regional and local planning and management. Participants’ background understanding of climate change science varied widely. In facilitated workshop discussions and pre-workshop surveys (Appendix B), participants indicated several factors that inhibit their ability to take action on addressing climate change effects with confidence, ranging from an uncertain understanding of future climate and ecological conditions, uncertainties in how to apply existing climate science to decision-making, and limited support from agency leadership and the public for taking local actions on climate change (Table S2).

**Workshop format**

While we adjusted some minor aspects of workshop format to address lessons learned during the earliest workshops, each workshop followed a generally consistent structure derived from the Adaptation for Conservation Targets (ACT) planning steps (Figure 2; Cross et al. In Press). For the Colorado, Arizona and Utah workshops, we integrated a few elements of TNC’s method for integrating climate change into Conservation Action Planning (TNC 2009) into the ACT approach. These slight modifications involved using terminology from the Conservation Action Planning method (e.g., “hypotheses of change” and “strategic adaptation actions”), and prompting participants to discuss ways in which human responses to climate change may affect the focal feature. Below is a generic version of a SWCCI workshop agenda that illustrates how we implemented the ACT steps in a workshop setting:

Day #1:

8:30 AM – 12:00 PM Plenary Session

- Welcome and overview of the Southwest Climate Change Initiative (SWCCI)
- Ice-breaker exercise where participants identify barriers to addressing climate change in their decision-making
- Introductory talks on relevant topics such as (varies from workshop-to-workshop depending on focal conservation features and availability of speakers):
  - Regional climate change effects: The known, the unknown, and the uncertain
  - Past and potential future trends in river/stream flows
  - Ecological consequences of climate change
  - Specific research about the effects of climate change on the workshop’s focal conservation features
- Presentation of the two future climate scenarios being considered during the workshop (see *Future Climate Scenarios* below)
- Overview of the Adaptation for Conservation Targets (ACT) framework and logistics for the remainder of the workshop

12:00 – 1:00 PM Lunch

1:00 – 5:00 PM Breakout Session #1 (one breakout group per conservation feature; each breakout group facilitated by two SWCCI core team members; breakout groups usually included about 15-20 participants)

- Objectives for breakout session #1 include ACT framework steps 1-3 (Fig. 2):
- Identify management goal: Begin with existing management goal for focal conservation feature as a starting point, or group discussion if no clearly articulated goal already exists. If multiple goals exist, merge into one goal or decide as a group which goal to focus on for this exercise.
- Refine the graphical conceptual model: Display a draft conceptual model developed by workshop steering team in advance of the workshop; allow participants to edit the draft as needed. The goal of this step is to identify climate and non-climate drivers the group considers to be the most important, and transparently display assumptions about relationships between drivers and the focal conservation feature.
- Assess potential effects of two future climate change scenarios: Group discussion to highlight potential climate change effects for future Climate Scenario #1 and Climate Scenario #2 (see *Future Climate Scenarios* below). Complete Table S3.

Day #2

8:30 AM – 12:00 PM Breakout Session #2 (same groups as on Day #1)

- Objectives for breakout session #2 include ACT framework steps 4-5, plus revisiting management goal and identifying information needs (Fig. 2):
- Identify management intervention points and strategic actions for climate change adaptation: Circle places on the graphical conceptual model where management actions may be possible. Brainstorm potential actions that can be taken at those intervention points towards achieving stated management goal in light of potential effects of future Climate Scenario #1. Consider whether those actions are also appropriate under future Climate Scenario #2, or whether different actions are needed under the second scenario. Complete Table S4.
- Review management goal: Consider whether management goal needs to be revised in light of potential effects of climate change and availability of adaptation actions.
- Identify priority actions: Identify several high-priority actions based on criteria such as: relative contribution to achieving the stated management goal; utility across both climate scenarios considered; and economic, social and political feasibility.
- List research and monitoring needs: Identify information needs discussed during both breakout sessions (collect these ideas as they come up on flip charts and then summarize at the end of Breakout Session #2).

12:00 – 1:00 PM Lunch

1:00 – 5:00 PM Plenary Session

- Report back from conservation feature breakout groups: highlight high priority strategic actions.
- Mini-breakout groups (stay in large plenary room) discuss barriers and opportunities for implementing priority strategic actions, followed by report-out and whole-group discussion. Indicate who (i.e., what people, agencies, organizations, etc.) needs to be involved to implement priority actions.
- Discussion on emerging themes, adaptation implementation and next steps.
- Participant feedback on workshop process and outcomes, and closing remarks.

**Pre-workshop webinars**

In response to comments by participants at the earliest workshops, we decided to reduce the amount of time spent on plenary presentations and help build participants’ capacity for more nuanced discussions of climate projections and their implications by offering participants at the last two workshops (for the Four Forest Restoration Initiative area and the Bear River basin) the opportunity to view a pre-workshop webinar on climate change science. During these webinars, Dr. Linda Mearns from the National Center for Atmospheric Research (NCAR) discussed climate system fundamentals, the basis for climate modeling, and uncertainties associated with projecting future climate. The webinar was offered twice before each workshop, and about 20 participants attended each one. The interactive format of the webinars allowed participants to pose questions and receive answers.

**Future climate scenarios**

We consulted with climate and hydrology experts from the region to customize future climate and hydrological scenarios for each workshop. We chose scenarios that allowed participants to examine uncertainties related to future precipitation changes relevant for each landscape, such as:

- A “warmer, drier future" versus a "warmer, drier future characterized by droughts of greater severity, magnitude, and extent" (see Jemez Mountains example below);
- A “warmer, drier future" versus a "warmer, wetter future" (see Bear River basin example below); or
- Seasonal differences, such as a “warmer, wetter winter” versus a “warmer, drier winter” (see Gunnison River basin example below).

The exact approach to developing scenarios varied across workshops, and depended to some degree on the team of scientists available to construct scenarios and provide climate model projections for parameters of interest to the selected conservation features. The first workshop—for the Jemez Mountains, New Mexico—included fairly general scenarios of changes in temperature, precipitation, and drought magnitude and frequency by 2100. In the subsequent three workshops, we worked more closely with climate experts to take a more sophisticated approach to assessing a range of climate model outputs when developing scenarios (see details below). We also added scenarios of future hydrology, and focused on a closer timeframe: 2040-2060. For the Four Forest Restoration Initiative workshop there were potentially confusing discrepancies between the climate scenario developed by a climate expert, and the hydrological scenarios developed by a hydrological modeler (see below for details). Based on this experience, we strongly recommend tight coordination between climate and hydrologic modelers to increase consistency in scenario development.

For all workshops, an attempt was made to include projections of climate and hydrologic information that addressed concerns related to the specific conservation features under consideration, such as seasonal precipitation amounts, or stream flows during particular times of year. Below are summaries of the future climate scenarios considered at each of the SWCCI workshops, and a few details on the development of those scenarios.

*Jemez Mountains, New Mexico:*

For the Jemez Mountains workshop, Dr. Todd Ringler from the Los Alamos National Laboratory reviewed the range of climate model projections for the region and developed two plausible scenarios for the end of the 21st Century (see Enquist et al. 2009 for additional details on scenario development).

Climate Scenario #1 = Increases in mean annual temperature between 2-4°C with increased drying, on average, and periodic extreme events in the first half of the century; precipitation reduced but skewed toward fewer larger events.

Climate Scenario #2: Increases in mean annual temperature between 2-6°C with increased drying, on average, and increased frequency of extreme events (e.g., episodic “mega” drought) by mid-century; note that drought has been a natural part of the SW for thousands of years. This scenario is essentially the same general temperature changes as in Scenario #1, but with only about 67% of its precipitation.

*Gunnison River basin, Colorado:*

For the Gunnison River basin workshop, Dr. Linda Mearns (NCAR) developed two climate change scenarios for 2040-2060 (Table S5) in coordination with Dr. Joe Barsugli from the Western Water Assessment at the University of Colorado (WWA-CU), who then used the climate scenarios to develop associated scenarios of hydrological change (Table S6) (see Neely et al. 2010 for additional details on climate and hydrological scenario development). Dr. Mearns based the climate scenarios on: (1) dynamically downscaled climate model projections using the high A2 greenhouse gas emissions scenario (IPCC 2000) from the North America Regional Climate Change Assessment Program (NARCCAP), and (2) global climate model projections using the medium-high A1B greenhouse gas emissions scenario (IPCC 2000) from the Intergovernmental Panel on Climate Change (IPCC) Fourth Assessment Report. Dr. Barsugli based the hydrological scenarios on modeling by Dr. Levi Brekke (U.S. Bureau of Reclamation, unpublished data), results from the Colorado River Water Availability Study by the Colorado Water Conservation Board, and other relevant sources.

*Four Forest Restoration Initiative area, Arizona:*

Dr. Linda Mearns (NCAR) developed future climate scenarios for 2040-2060 for the Four Forest Restoration Initiative area (Table S7) and Seshadri Rajagopal and colleagues at the University of Arizona provided preliminary hydrologic projections for the Salt and Verde River Basins for 2039-2068 (Table S8) (see Smith et al. 2011 for additional details on climate and hydrological scenario development). Dr. Mearns based the climate scenarios on: (1) dynamically downscaled climate model projections using the high A2 greenhouse gas emissions scenario from the NARCCAP, and (2) global climate model projections using the medium-high A1B greenhouse gas emissions scenario from the IPCC Fourth Assessment Report. Rajagopal et al. used the University of Washington's Variable Infiltration Capacity (VIC) model (Liang et al. 1994; Gao et al. 2009) under three greenhouse gas emissions scenarios (B1, A1B, A2) to develop the hydrological scenarios. Rajagopal et al. based their preliminary analysis on the ensemble results from three climate models that demonstrated the most faithful reproduction of historic temperature and precipitation variations, and of key atmospheric circulation features, such as the El Niño-Southern Oscillation and the North American monsoon (Dominguez et al. 2010).

The different choices of climate models resulted in some divergence between Dr. Mearns' summer precipitation projections (both of which were decreasing) and the precipitation inputs to Rajagopal et al.'s hydrological model (all of which showed increasing summer precipitation); nevertheless, the streamflow projections were considered credible and were used to illustrate uncertainties associated with projecting future conditions. In response to these discrepancies, Dr. Mearns developed a third “alternate” (yet plausible) scenario that included intermediate levels of warming and less dramatic decreases in annual precipitation due to an increase in summer precipitation (Smith et al. 2011). One of the breakout groups had sufficient time to explore the consequences of this third scenario, but the other two groups focused primarily on the two climate scenarios detailed in Table S7.

*Bear River basin, Utah/Wyoming/Idaho:*

For the Bear River basin workshop, Dr. Linda Mearns (NCAR) developed two climate change scenarios for 2040-2060 (Table S9) in collaboration with Dr. Joe Barsugli (WWA-CU), who developed associated scenarios of hydrological change (Table S10) (see Degiorgio et al. 2010 for additional details on climate and hydrological scenario development). Dr. Mearns based the climate scenarios on: (1) dynamically downscaled climate model projections using the high A2 greenhouse gas emissions scenario from the NARCCAP, and (2) global climate model projections using the medium-high A1B greenhouse gas emissions scenario from the IPCC Fourth Assessment Report. Dr. Barsugli created the hydrological scenarios by running the two scenarios of temperature and precipitation changes through the VIC hydrologic model (Liang et al. 1994; Gao et al. 2009), using parameters appropriate to the Bear River. Dr. Barsugli’s analyses only considered natural flows that are unaltered by diversions and reservoir storage, and do not apply to reaches of the river where groundwater interactions are important.

**Literature cited**

**Table S1.** Agencies and organizations that participated in each Southwest Climate Change Initiative (SWCCI) workshop (not including core SWCCI organizations: the Nature Conservancy, Climate Assessment for the Southwest/University of Arizona, Wildlife Conservation Society, Western Water Assessment/University of Colorado, University of Washington, and National Center for Atmospheric Research).

| Jemez Mountains, New Mexico | Gunnison River basin, Colorado | Four Forest Restoration Initiative, Arizona | Bear River basin, Utah/Wyoming/Idaho |
| --- | --- | --- | --- |
| Bureau of Indian Affairs  Forest Guild  Four Corners Institute  Highlands University  Jemez Pueblo  Los Alamos National Laboratory  National Park Service  New Mexico Department of Game and Fish  New Mexico Energy, Resources and Natural Resources Department  New Mexico State Forestry  Santa Ana Pueblo  Santa Clara Pueblo  U.S. Fish and Wildlife Service  U.S. Forest Service  U.S. Geological Survey  Valles Caldera National Preserve | Bureau of Land Management  Colorado Division of Wildlife  Colorado Natural Heritage Program  Colorado Water Conservation Board  Gunnison County  High Country Citizens Alliance  Mountain Studies Institute  National Park Service  Natural Resources Conservation Service  Rocky Mountain Biological Laboratory  Upper Gunnison River Conservancy District  U.S. Forest Service  U.S. Fish & Wildlife Service  Western State College | Arizona Game and Fish Department  Arizona State Forestry Division  Grand Canyon Trust  National Park Service  Northern Arizona University  U.S. Fish and Wildlife Service  U.S. Forest Service | Bear River Association of Governments  Bridgerland Audubon  Bureau of Land Management  Cache County  Inland Sea Shorebird Reserve Manager (Kennecott)  National Oceanic and Atmospheric Administration  PacifiCorp  Trout Unlimited  U.S. Fish & Wildlife Service  U.S. Forest Service  Utah Division of Wildlife Resources  Utah State Geologic Survey  Utah State University  Weber State University  Wild Utah Project  Wyoming Game and Fish |

**Table S2. Challenges to taking action on climate change adaptation indicated by participants at the Bear River basin workshop during a facilitated workshop discussion (Degiorgio et al. 2010) and pre-workshop surveys (Appendix B).**

| Institutional and public attitudes toward climate change and uncertainty:   - The private sector is risk averse and does not tolerate uncertainty well; - Uncertainty leads to lack of support for making planning changes; - In Utah, the prevalent attitude toward climate change is one of skepticism: agency and private sector denial of the results of climate change science and resistance to uncertainty results in constraints on data collection and even data use. |
| --- |
| Policy and political will:   - There is a lack of policy experimentation and innovation, political will to change, and a lack of resources for change. - Current planning processes make it difficult to incorporate new management approaches. - Land management decisions institutionalized by land management agencies and private land owners have not yet incorporated climate change into their decision processes. |
| Basic and applied science:   - Inadequate characterization of resilience: What does it mean to different organizations? - Inadequate information on the combined and interacting effects of climate change and other factors: How will these play out? - Inadequate knowledge of how climate change will affect ecosystems and ecosystem processes, and an inadequate ability to conceptualize change. - Inadequate information on ecosystem trajectories. - Lack of information on how climate change will affect individual species, hydrology, and Bear River water resources. - Information about climate change is too uncertain. |
| Management, decision, and risk science:   - Information about climate change is available, but it is unclear how best to use it. - Lack of information on whether any of the proposed strategies for coping with climate change will work. - Lack of information on whether current management methods will be appropriate for the future. |

* While these responses are specific to the participants at the Bear River basin workshop, many similar challenges were discussed by participants at the other Southwest Climate Change Initiative workshops (see Enquist et al. 2009; Neely et al. 2010; Smith et al. 2011 for responses from participants at other workshops).

**Table S3.** Template for table used to document participant discussions about climate change effects (also called ‘Hypotheses of Change’).

| Conservation Feature: ________(fill-in conservation feature here)_________________________________ ­­­­­­  Future Climate Scenario #1: ___(fill-in climate scenario #1 information here)________________________  Future Climate Scenario #2: ___(fill-in climate scenario #2 information here)________________________ | | |
| --- | --- | --- |
| Key Climate-Influenced Drivers/Effects  (e.g., Physical, Ecological, Social, Economic) | Observed & Projected  Climate Change Impact^a^  (i.e., Hypotheses of Change) | Comments, Notes, Sources^b^ |
|
|  |  |  |
|  |  |  |
|  |  |  |
|  |  |  |
|  |  |  |
|  |  |  |
|  |  |  |
|  |  |  |
|  |  |  |

^a^ Indicate which future climate scenario the impact applies to: “S1” = Scenario #1 only, “S2” = Scenario #2 only, or “S1 & S2” = both.

^b^ Time permitting, indicate relatively likelihood and severity of impact.

**Table S4.** Template for table used to document participant discussions about strategic actions to address climate change effects.

| Conservation Feature: _____(fill-in conservation feature here)_ ____________________  Management Goal: _______(fill-in management goal here)__________________________ | | | | |
| --- | --- | --- | --- | --- |
| Observed & Projected  Climate Change Impact  (Hypotheses of Change) ^a^ | Intervention Point | Strategic Actions for  Climate Scenario #1  (Planning Horizon = __ years) ^b^ | Strategic Actions for  Climate Scenario #2  (Planning Horizon = __ years) ^b^ | Notes^c^ |
|  |  |  |  |  |
|  |  |  |  |  |
|  |  |  |  |  |
|  |  |  |  |  |
|  |  |  |  |  |
|  |  |  |  |  |
|  |  |  |  |  |
|  |  |  |  |  |
|  |  |  |  |  |
|  |  |  |  |  |

^a^ Transferred from Table S3.

^b^ Indicate the planning horizon under consideration (e.g., actions to be taken in the next 5-10 years, or some other time horizon).

^c^ Time permitting, indicate level of urgency or priority of action, and opportunities to implement the action.

**Table S5.** Future climate scenarios for 2040-2060 for the Gunnison River basin (Colorado) workshop.

| Season | Climate Scenario #1 | | Climate Scenario #2 | |
| --- | --- | --- | --- | --- |
|  | Temperature °C | Precipitation % | Temperature °C | Precipitation % |
| Annual | +2.0-3.0 | ~0.0 | +3.0-4.0 | -10.0 |
| Winter | +2.0 | +15.0 | +3.0 | ~0.0 |
| Spring | +2.5 | -12.0 | +3.0 | -15.0 |
| Summer | +3.0 | -15.0 | +4.0 | -20.0 |
| Fall | +2.5 | +4.0 | +3.0 | -10.0 |

**Table S6.** Hydrologic implications of climate scenarios (Table S5) for the Gunnison River basin (Colorado) workshop.

| Parameter | Hydrological implications of  Climate Scenario #1 | Hydrological implications of  Climate Scenario #2 |
| --- | --- | --- |
| Streamflow amount | Annual natural stream flow will decrease under a scenario of increased temperature, even if precipitation remains the same. The shift of precipitation from summer to winter somewhat counteracts the drying tendency somewhat leading to a moderate decrease (5-10%) in annual flows. | Decrease in precipitation and increase in temperature both act to reduce annual stream flow totals. Projected stream flow decreases are in the range of 20-25%. |
| Snowpack accumulation and melt | Warming temperatures lead to a later accumulation of snow in the fall, and an earlier snowmelt in the spring. However, because of the increased precipitation in winter, and the generally cold, high-elevation nature of the Upper Gunnison Basin, the mid-winter snowpack may be similar to the present. | Warming temperatures lead to a later accumulation of snow in the fall, and an earlier snowmelt in the spring. Because this likely represents a hot/dry scenario for much of the West, the potential exists for more frequent dust deposition events, which also may lead to an earlier melt and to reduced water yield from the snowpack. |
| Streamflow timing | Snowmelt-driven stream flow will peak about 7 days earlier, on average, in the spring. | Snowmelt-driven stream flow will peak about 14 or more days earlier, on average, in the spring. |
| Soil moisture | The earlier melt along with decreased summer precipitation and increased summer temperatures results in significantly lower amounts of water stored in the soils during summer. | The much earlier melt, along with decreased summer precipitation and increased summer temperatures, results in extremely low amounts of water stored in the soils during summer and fall |

**Table S7.** Future climate scenarios for 2040-2060 for the Four Forest Restoration Initiative area (Arizona) workshop.

| Season | Climate Scenario #1 | | Climate Scenario #2 | |
| --- | --- | --- | --- | --- |
|  | Temperature °C | Precipitation % | Temperature °C | Precipitation % |
| Annual | 2.0 | -14 | 3.4 | -25 |
| Winter | 1.3 | -13 | 3.5 | -14 |
| Spring | 1.8 | -16 | 3.0 | -35 |
| Summer | 2.5 | - 8 | 4.0 | -35 |
| Fall | 2.5 | -24 | 2.5 | -25 |

**Table S8.** Potential hydrological changes for the Four Forest Restoration Initiative area (Arizona) workshop*.

| Parameter | Hydrological implications of projected changes in temperature and precipitation |
| --- | --- |
| Runoff amount | Salt River annual runoff decreases in hydrological model results averaged for each emissions scenario, from -9.3% (B1) to -23.5% (A1B). |
| Snowpack accumulation and melt | Lower peak snowpack accumulation (approximately 50% lower than observed peak accumulation). |
| Runoff timing | Warming temperatures lead to earlier snowpack melt and earlier runoff timing. |
| Summer flows | Summer flows increase under each emission scenario, with a range of +3.6% (A1B) to +13.5% (A2). |
| Winter flows | Winter flows decrease under each emission scenario, with a range of -13.8% (B1) to -28.9% (A1B); the frequency of rain on snow events may increase. |

* See Appendix A text for description of how these scenarios were developed, and divergence between the climate models used to develop the Four Forest Restoration Initiative climate scenarios (Table S7) and the climate models used to develop the hydrological projections described here.

**Table S9.** Future climate scenarios for 2040-2060 for the Bear River basin (Utah/Wyoming/Idaho) workshop.

| Season | Climate Scenario #1 | | Climate Scenario #2 | |
| --- | --- | --- | --- | --- |
|  | Temperature °C | Precipitation % | Temperature °C | Precipitation % |
| Annual | 3.5 | +1.6 | 2.7 | -3 |
| Winter | 2.5 | +13 | 2.7 | -5 |
| Spring | 3.5 | -6 | 2.0 | +10 |
| Summer | 4.5 | -15 | 3.0 | -20 |
| Fall | 3.5 | 0 | 3.0 | +3 |

**Table S10.** Hydrologic implications of climate scenarios (Table S8) for the Bear River basin (Utah/Wyoming/Idaho) workshop.

| Parameter | Hydrological implications of  Climate Scenario #1 | Hydrological implications of  Climate Scenario #2 |
| --- | --- | --- |
| Runoff amount | 5-18% decrease in annual runoff | 5-13% decrease in annual runoff |
| Snowpack accumulation and melt | Later fall accumulation; 10-15% lower peak accumulation; earlier spring melt ~2-4 weeks | Later fall accumulation; 15-10% lower peak accumulation; earlier spring melt ~2-4 weeks |
| Runoff timing | Earlier by 1-3 weeks | Earlier by 1-2 weeks |
| Summer flows | Low flows = -10%  High flows = -25% | Low flows = -15%  High flows = -50% |
| Winter flows | 30-50% increase, due to more rain events | 30-50% increase, due to more rain events |
