# Supplementary material for: Accelerating Adaptation of Natural Resource Management to Address Climate Change
Source: Conserv Biol. 2012 Oct 30;27(1):4–13. doi: 10.1111/j.1523-1739.2012.01954.x (PMC3562478; doi:10.1111/j.1523-1739.2012.01954.x)
Supplement: Supplementary file 2 [file cobi0027-0004-SD2.doc]

**Supporting Information Appendix S2.**

**WORKSHOP SURVEYS**

Preworkshop Participant Surveys

Pre-workshop surveys were distributed to participants before each of the last two workshops—for the Four Forest Restoration Initiative area and the Bear River basin—to assess invited participants’ interests and needs related to climate change and adaptation, and to inform the design of a pre-workshop webinar (Appendix A). The survey below was distributed to invited participants in advance of the Four Forest Restoration Initiative area workshop. For the pre-workshop survey for the Bear River basin workshop, we only asked questions #1-6. Results for the two pre-workshop surveys are available on request.

*Introduction: purpose and benefits*

The purpose of this survey is to assess participants’ knowledge, interests and needs related to climate change and climate adaptation. Your responses will help the organizers design a more effective workshop.

*Confidentiality*

Your responses will remain anonymous, though we may make a summary of all responses available to participants.

*Time commitment*

Completing the survey should take 10-15 minutes.

1. Please identify your organizational affiliation.

- US Forest Service
- National Park Service
- US Fish and Wildlife Service
- Arizona State Forestry Division
- Northern Arizona University
- Other academic institution
- Non-profit conservation organization
- Other: __________

1. Please identify your profession.

- Forester
- Planner
- Wildlife biologist/manager
- Landscape ecologist
- Water/watershed manager
- Hydrologist
- University scientist/researcher
- Conservation activist
- Other: _______________________

1. Which geographic scale do your management and planning decisions affect?

- Project (tens to thousands of acres)
- Management sub-unit (e.g., ranger district, or thousands of acres)
- Large management unit (e.g., national forest, or hundreds of thousands to millions of acres)
- Region (multiple non-contiguous management units across a geographic region)
- None: I am not a planner or manager

1. Do you have all the information you need to make well-informed natural resource management decisions or plans in the face of climate change? (Rate from 1 to 5, where 1=excellent information and 5= far too little information)
2. What information do you most urgently need to address climate change in your planning and management decisions?
3. Which of the following are the greatest challenges for you in developing climate change adaptation plans and projects in northern Arizona? (Choose all that apply.)

- Not enough information available about actual and projected effects of climate change in my area.
- Information about climate change is too uncertain for me to use in my work.
- Information about climate change is available, but it is unclear how I can best use it.
- Too many other demands on my time; dealing with climate change is not urgent.
- Current planning processes make it difficult to incorporate new management approaches.

1. Which subject areas would you find most interesting and useful for exploration at the workshop and pre-workshop webinars? (Rank each option from 1 [not useful] to 5 [very useful])

- The basics of climate science: functioning of the global and regional climate, major influences on climate from natural and human sources, and how climate models work.
- Climate trends in the southwestern U.S. over past millennia and the recent past (the last half century).
- Climate change scenarios for northern Arizona: projections for the next several decades.
- Observed and plausible future effects of climate change on the species and ecosystems of northern Arizona.
- Observed and plausible future effects of climate change on the hydrology of northern Arizona.
- How vegetation modeling is used to develop scenarios of potential effects of climate change on ecosystems.
- Methods for assessing the vulnerability of species to climate change.
- How to use climate science and ecological science to develop adaptation strategies for species, habitats and ecosystems.
- Sources of uncertainty in climate change models, scenarios, and dissemination of information.
- Other:_______________________

1. Have you received academic or professional training in meteorology or climatology? (Yes or no.)
2. How likely are you to use the following sources of climate change information in your work?

(Rate from 1-5, where 1=very unlikely, 5-very likely.)

- Television
- Newspaper
- Blogs
- Colleagues
- Magazine or popular journal
- Scientific journals
- Federal or state supported web sites, data portals, or publications
- University web sites, data portals, or publications
- Non-governmental organization (NGO) web sites, data portals, or publications

1. The difference between weather and climate is: (Choose one.)

- Weather describes short-term (minutes to days) variations in the atmosphere, whereas climate describes long-term variations (months or longer).
- Climate only describes the average long-term conditions, whereas weather describes the variations around the average long-term conditions.
- Weather is predictable, but climate is not.
- Weather includes more variables, like moisture and wind, whereas climate just focuses on temperature and precipitation.

1. Climate variability is: (Choose all that apply)

- A consistent long-term trend caused by natural factors
- The natural year-to-year changes in climate
- Increasing because of the greenhouse effect
- A part of climate change

1. Which of the following has the least influence on global climate? (Choose one.)

- Variation in solar radiation
- Greenhouse gases
- The ozone hole
- Vegetation and land use change
- Volcanic eruptions
- I don’t know

1. The greenhouse effect is: (Choose one)

- Produced by the accumulation of certain atmospheric gases
- A warming effect produced solely by pollution
- Caused by the ozone hole
- I don’t know

1. True or False? (Note whether each statement is true or false.)

- The global climate can be changed by human-caused emissions of greenhouse gases.
- The decreasing rate of global temperature increases in the last few years means that global warming is slowing down.
- If emissions of greenhouse gases stopped today, then global warming would stop and temperatures would quickly decline.
- The magnitude and direction of observed climate change are not the same at all locations on Earth.

1. Global circulation models (GCM) have been used extensively by the Intergovernmental Panel on Climate Change (IPCC) and other scientific authorities to project changes in the climate over the next several decades. Which of the following statements is TRUE of these climate models:

- The higher the resolution of the model results (e.g., 1 km rather than 10 km), the more accurate and less uncertain these projections are likely to be.
- The accuracy and uncertainty of the model results is independent of their spatial resolution.

1. Which of the following best characterizes climate in northern Arizona over the past 30 years? (Choose one)

- Average annual temperature and total annual precipitation have increased.
- Average annual temperature has increased, and total annual precipitation has decreased.
- Average annual temperature have increased, but no change in average annual precipitation has been detected
- No change in average annual temperature or total annual precipitation has been recorded.
- I don’t know

1. Please identify three likely impacts of climate change on natural resources (forests, grasslands, streams, etc.) in northern Arizona.

Post-Workshop Exit Surveys

After the first SWCCI workshop (focused on the Jemez Mountains), we decided to gather participants’ assessments of the success of presentations, breakout sessions, plenary discussions and the workshop as a whole, in order to improve the design of future workshops. Exit surveys were therefore distributed to all participants as they left the Gunnison River basin and Four Forest Restoration Initiative area. The survey below was distributed to participants at the close of the Gunnison River basin workshop. To streamline the exit surveys **f**or the Four Forest Restoration Initiative area workshop, we only included a single column with a slightly different question: “This presentation or activity increased my ability to understand climate change and how I can integrate it into my management/conservation work**”**. We also removed a question asking about the usefulness of a session where we articulate priority research and information needs since we did not hold a special discussion on that topic during the Four Forest Restoration Initiative workshop. Several results from the Gunnison River basin exit surveys are presented in Figures S1-S13 (a total of 35 surveys were returned). Although there was a lower response rate on exit surveys for the Four Forest Restoration Initiative workshop (only 17 surveys were returned), those surveys that were returned yielded similar feedback as for the Gunnison workshop (Figures S14-S25).

**Gunnison River basin exit survey questions:**

#### I am a:

#### Public Agency Natural Resource Planner or Manager

#### Public Agency or Academic Scientist

#### Breakout Session Facilitator

#### Breakout Session Notetaker

#### The Nature Conservancy or other NGO Employee

#### Other: ________________________________________________________

1=Not at All 2=Marginal 3=Neutral 4=Mostly 5=Absolutely

|  | This presentation or activity provided valuable information that ****enhanced my understanding of climate adaptation issues**** | This presentation or activity increased my ability to incorporate climate change impacts into my management or conservation work |
| --- | --- | --- |
| Day 1 – Wednesday, December 2 | Not  at all Absolutely | Not  at all Absolutely |
| 1. Welcome and opening presentations by Sullivan, McCarthy and Garfin | 1 2 3 4 5 | 1 2 3 4 5 |
| 1. Climate change science presentations by Mearns, Billick, Inouye and Barsugli | 1 2 3 4 5 | 1 2 3 4 5 |
| 1. Overview of conservation adaptation planning and explanation of adaptation framework by Cross and Garfin | 1 2 3 4 5 | 1 2 3 4 5 |
| 1. Break-outs: *management objectives, models & climate change impacts* | 1 2 3 4 5 | 1 2 3 4 5 |
| Day 2 – Thursday, December 3 |  |  |
| 1. Break-outs: *strategic actions, research and monitoring needs* | 1 2 3 4 5 | 1 2 3 4 5 |
| 1. Full group presentation and discussion: *priority strategic actions* | 1 2 3 4 5 | 1 2 3 4 5 |
| 1. Full group discussion: *monitoring & research priorities* | 1 2 3 4 5 | 1 2 3 4 5 |
| 1. Panel discussion: *implementing workshop recommendations* | 1 2 3 4 5 | 1 2 3 4 5 |
| 1. Workshop summary and closing remarks by McCarthy and Sullivan | 1 2 3 4 5 | 1 2 3 4 5 |
| Overall Workshop |  |  |
| 1. I have a better understanding of the impacts of climate change on the Gunnison Basin. | 1 2 3 4 5 |  |
| 1. The framework presented is useful for developing climate adaptation strategies. | 1 2 3 4 5 |  |
| 1. The presenters were knowledgeable & well prepared. | 1 2 3 4 5 |  |
| 1. The facilitators were knowledgeable & well prepared. | 1 2 3 4 5 |  |
| 1. The workshop was the right size. | 1 2 3 4 5 |  |
| 1. Overall, I have a better understanding of the resources that are available and how to incorporate climate adaptation strategies into my conservation work. | 1 2 3 4 5 |  |

| The most helpful/useful things from this Climate Change Adaptation Workshop to me were: |
| --- |
|  |
|  |
| For applying this process in the future, I would recommend the following additions/changes: |
|  |
|  |
|  |
| ADDITIONAL COMMENTS: |
|  |
|  |
|  |


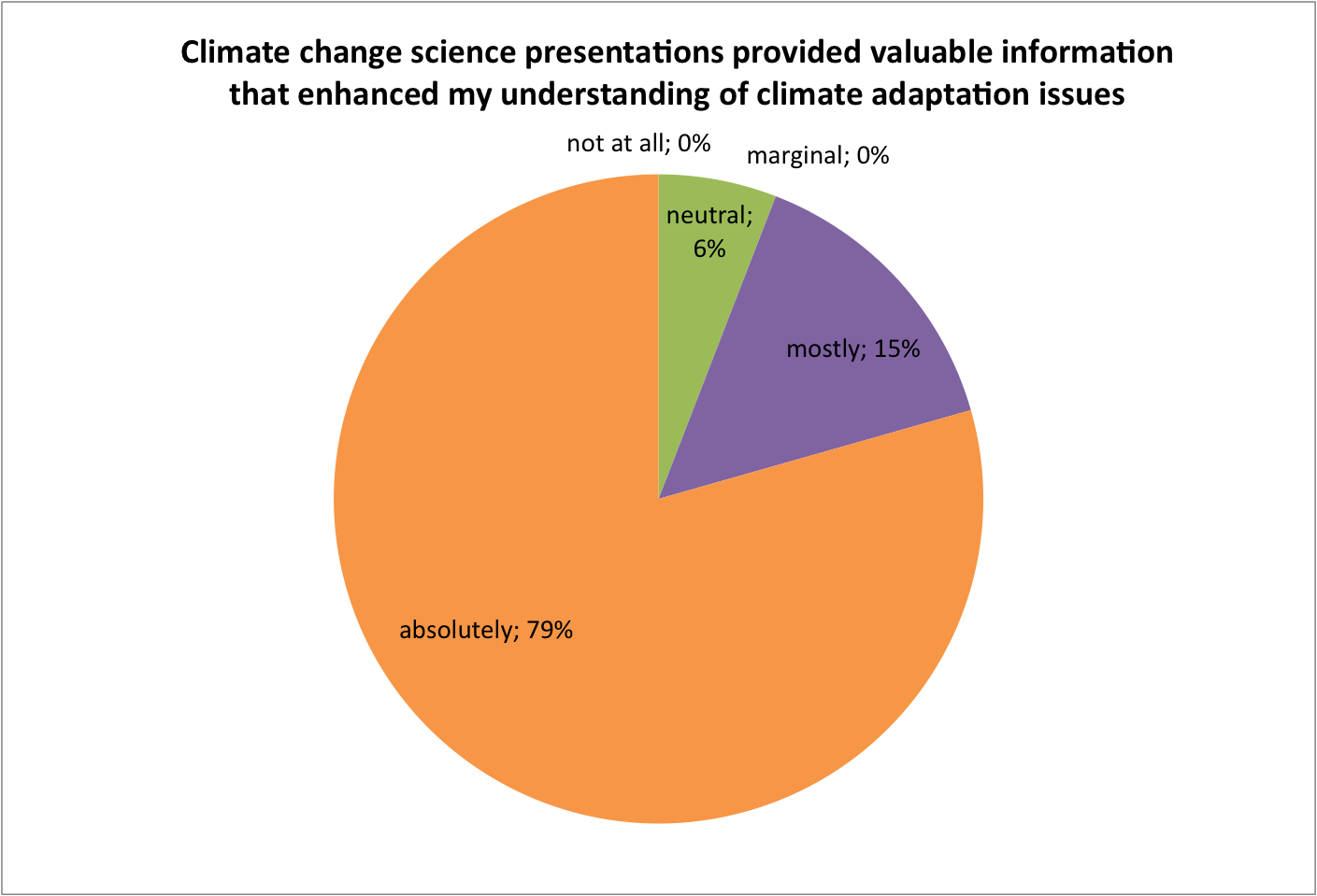


Figure S1. Gunnison River basin workshop participants’ opinions about the introductory climate change presentations (N = 34).


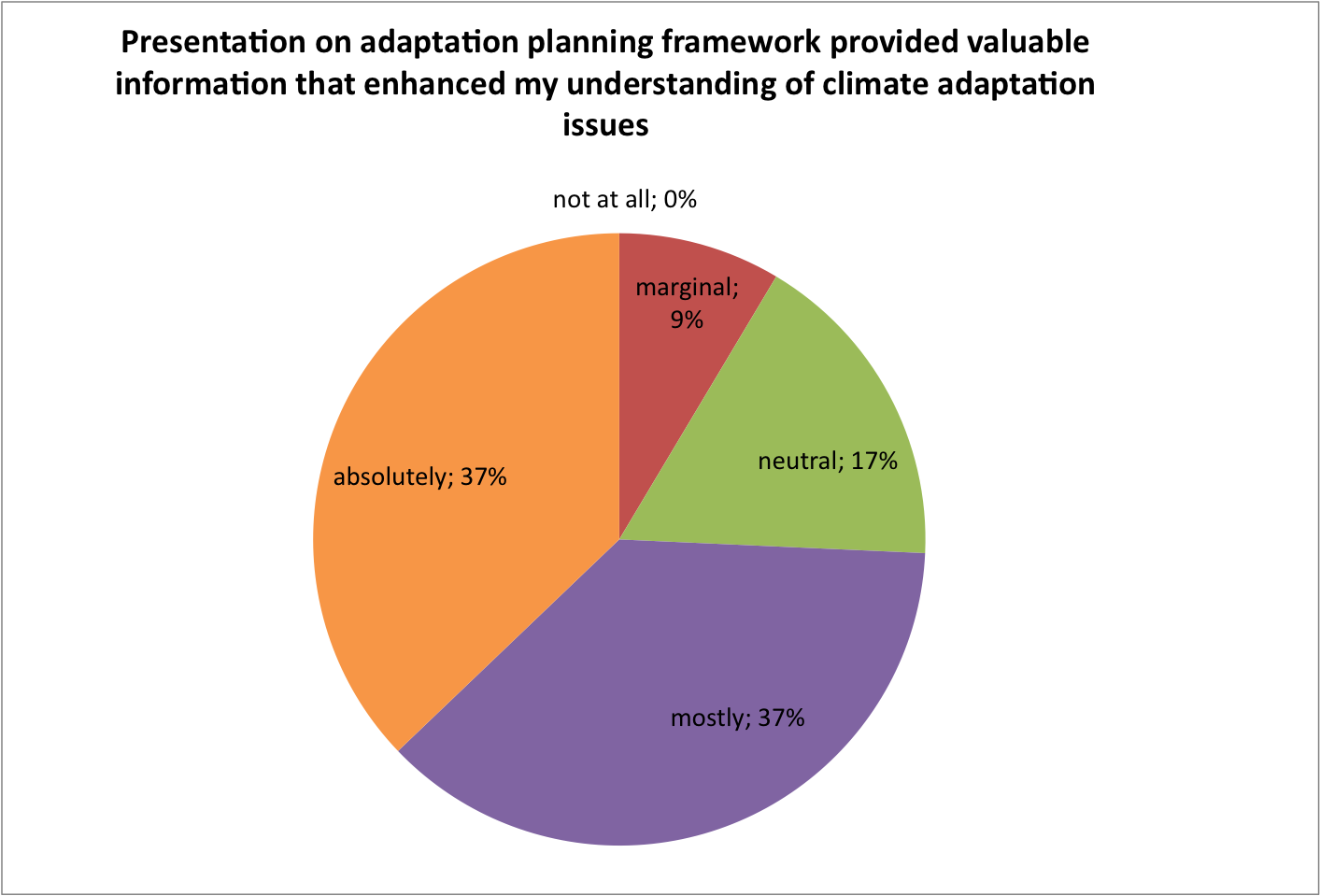


Figure S2. Gunnison River basin workshop participants’ opinions about the introductory presentation on the Adaptation for Conservation Targets (ACT) planning framework (N = 35).


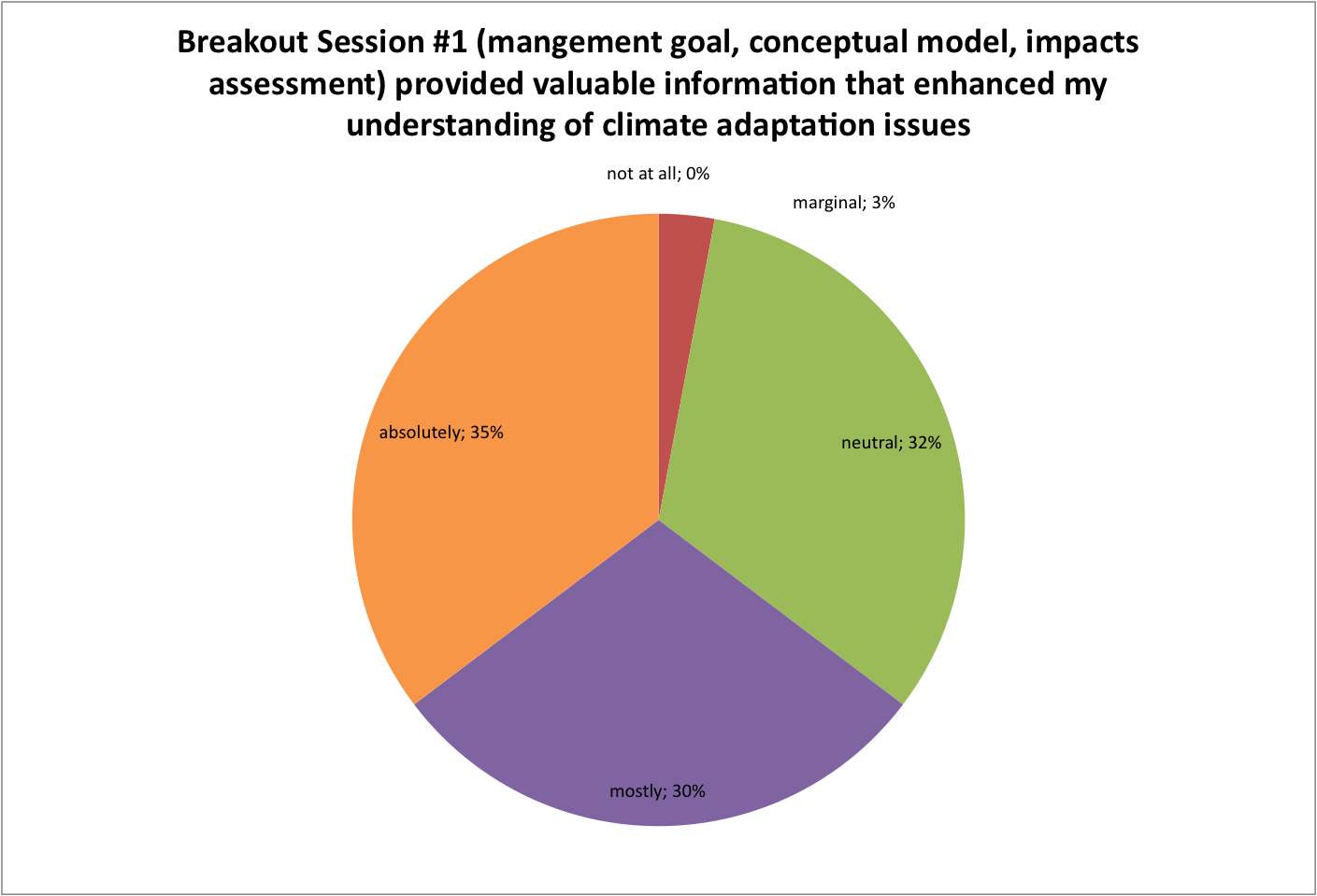


Figure S3. Gunnison River basin workshop participants’ opinions about the first breakout session when small groups specified management goals, refined conceptual models, and assessed climate change effects for selected species, ecological processes or ecosystems (N = 34).


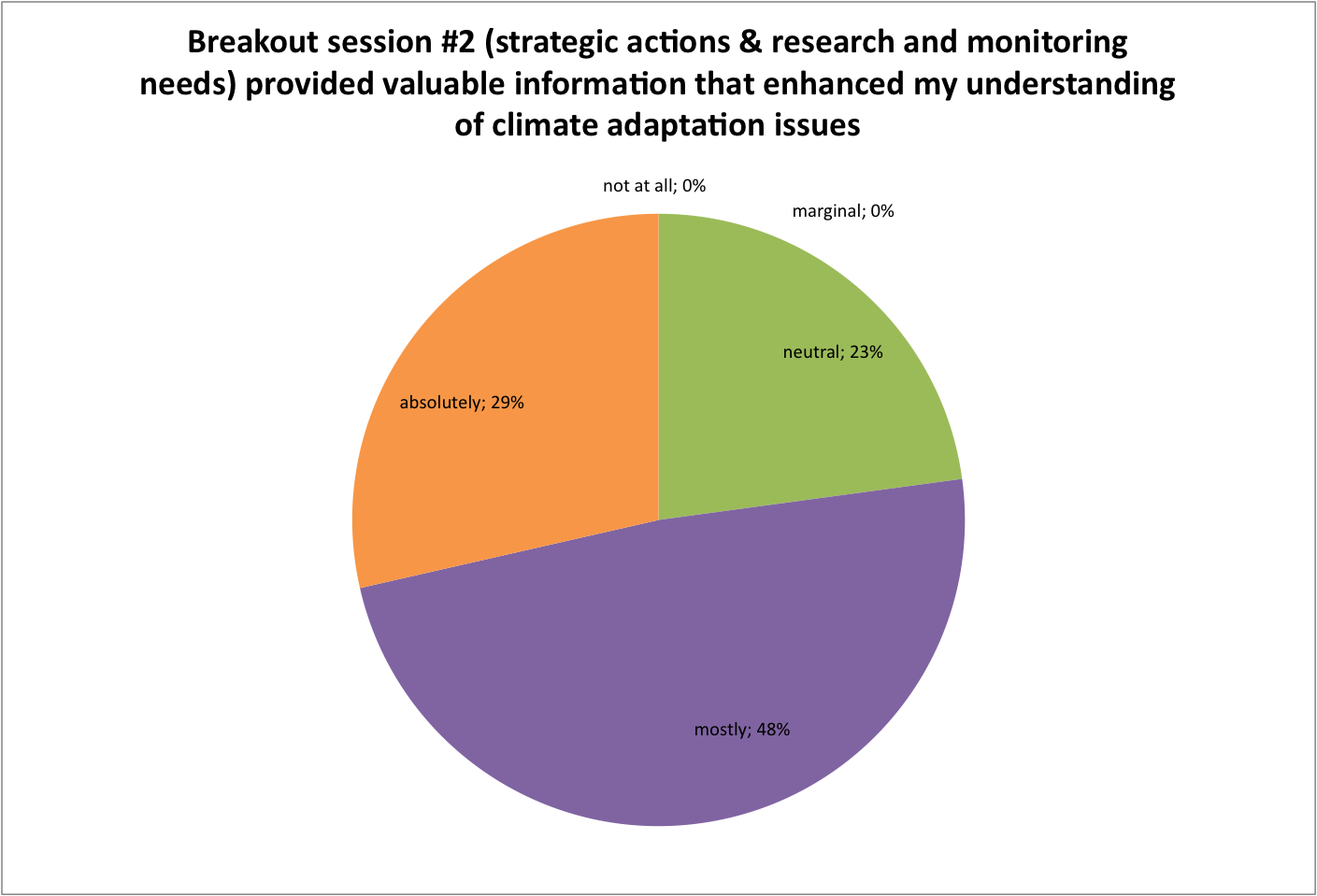
Figure S4. Gunnison River basin workshop participants’ opinions about the second breakout session when small groups identified strategic adaptation actions, revisited management goals, and identified research and monitoring priorities (N = 35).


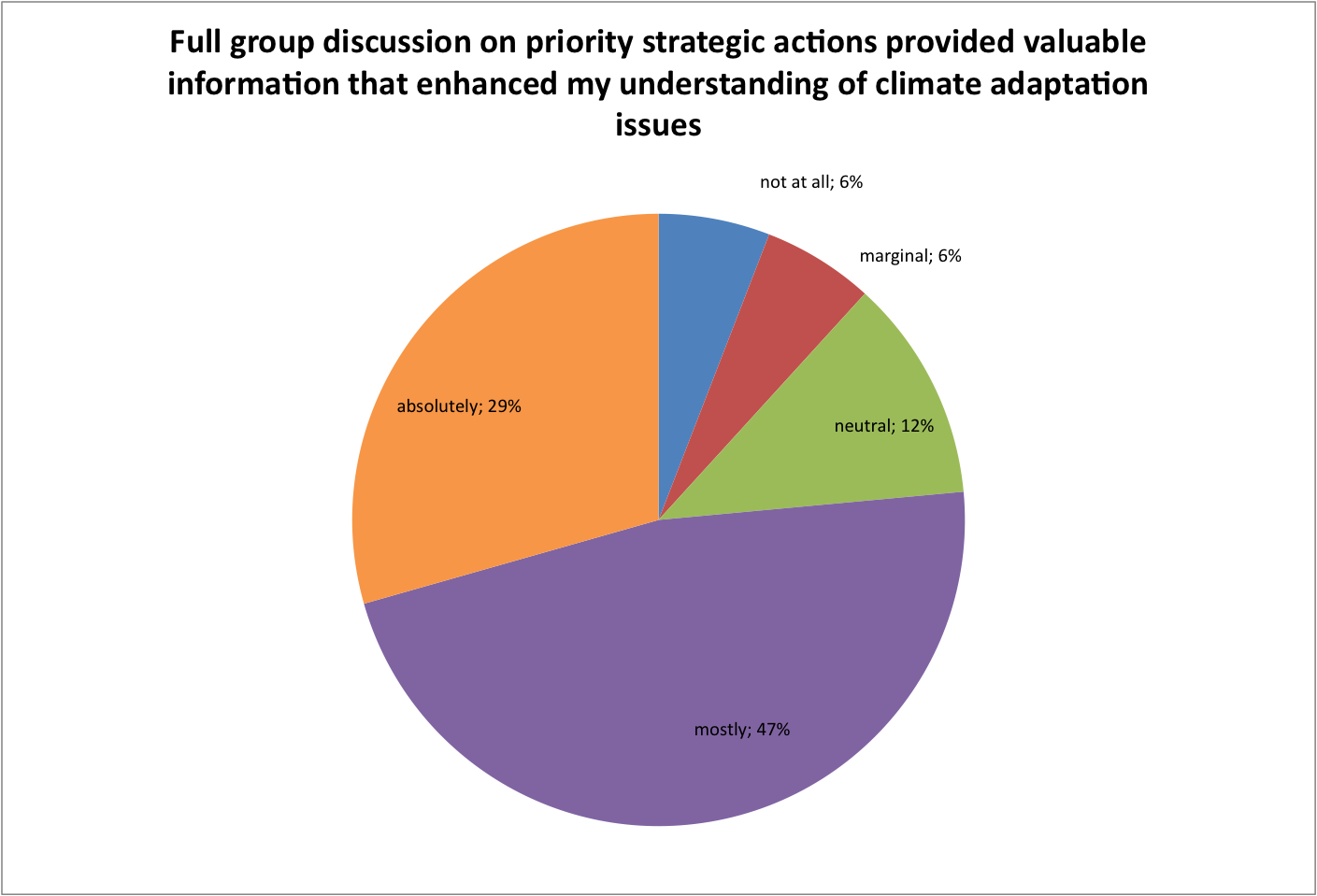
Figure S5. Gunnison River basin workshop participants’ opinions about the full-group discussions about priority strategic adaptation actions from each breakout session (N = 34).


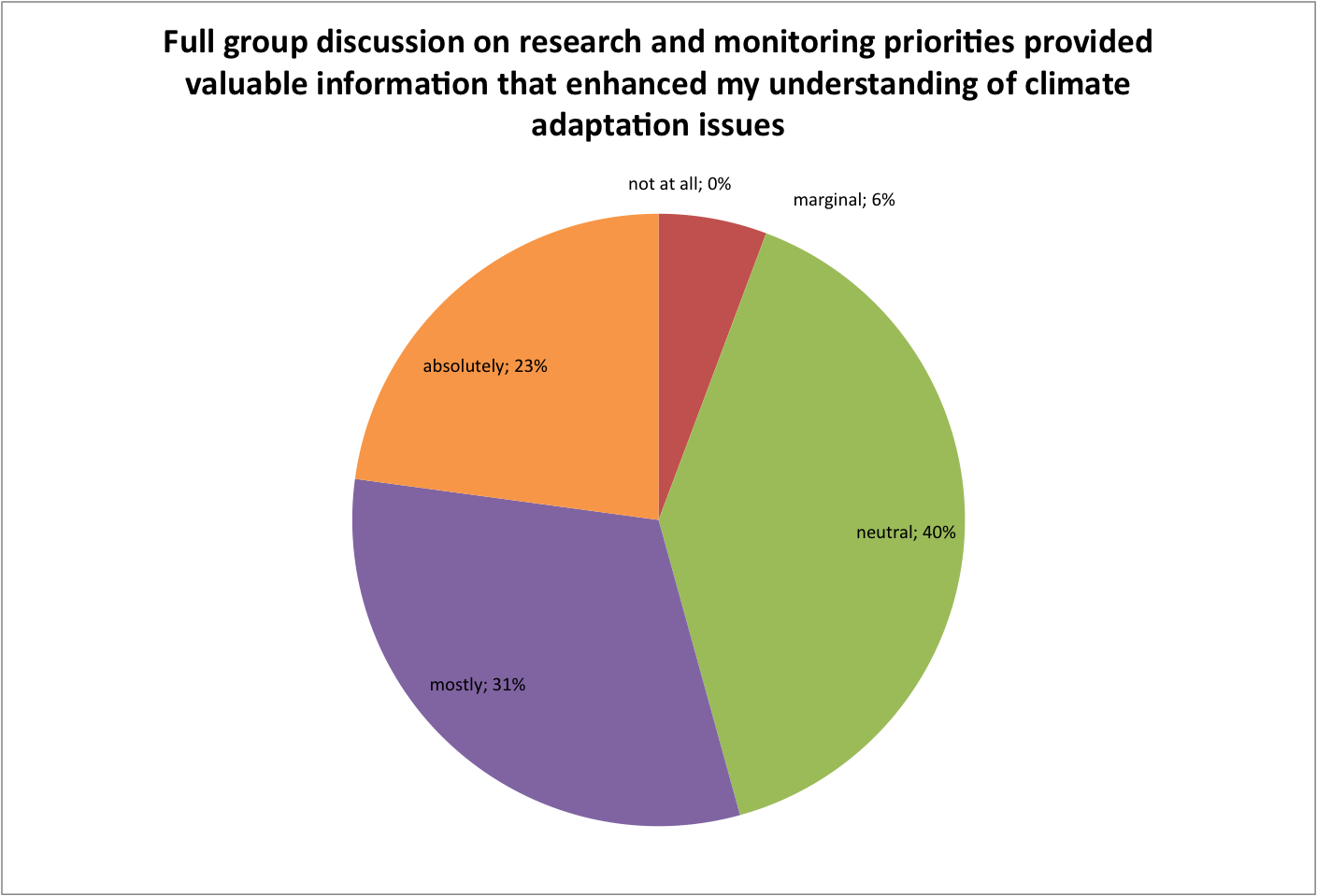


Figure S6. Gunnison River basin workshop participants’ opinions about the full-group discussion on research and monitoring priorities (N = 35).


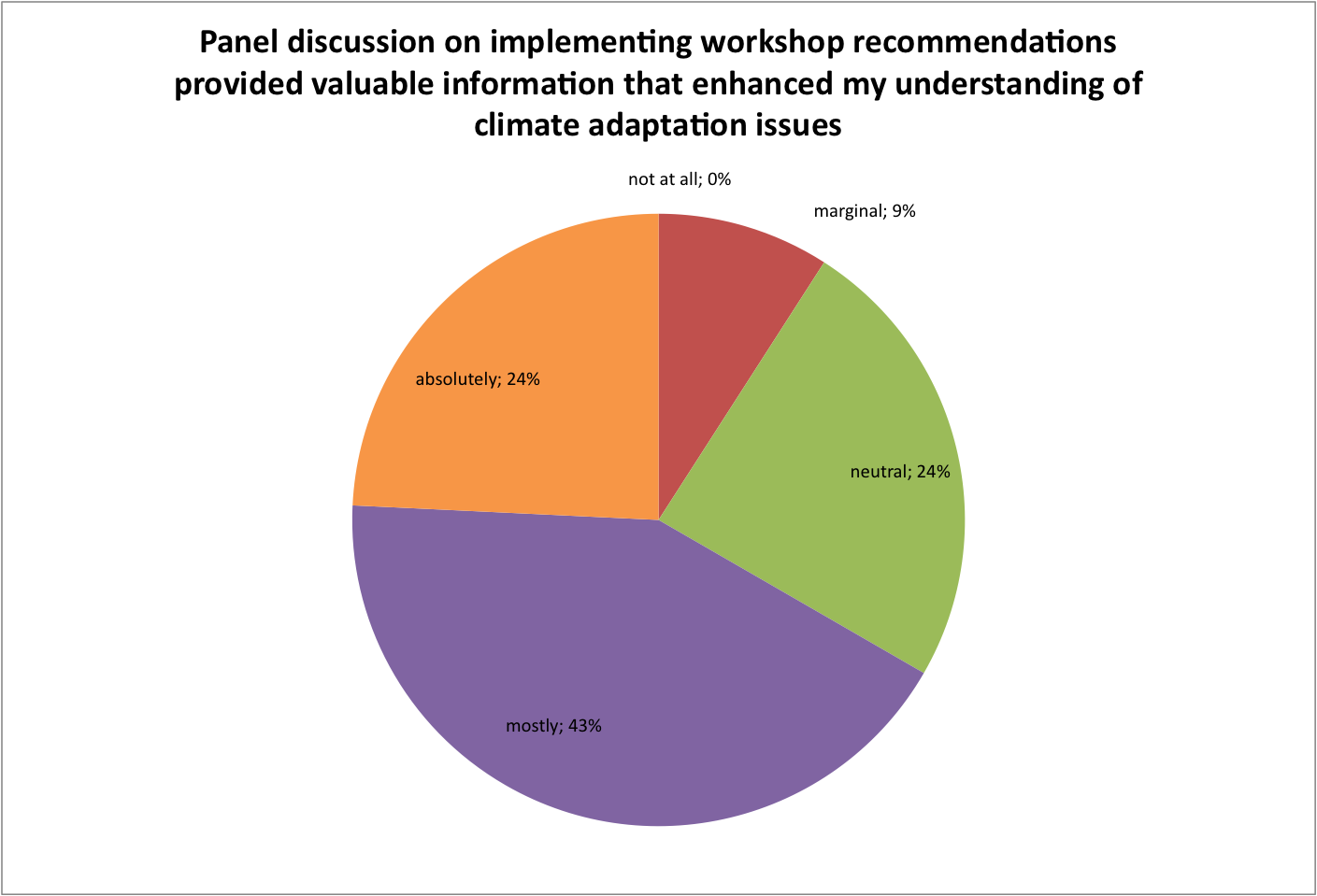


Figure S7. Gunnison River basin workshop participants’ opinions about the panel discussion on implementing workshop recommendations (N = 33).


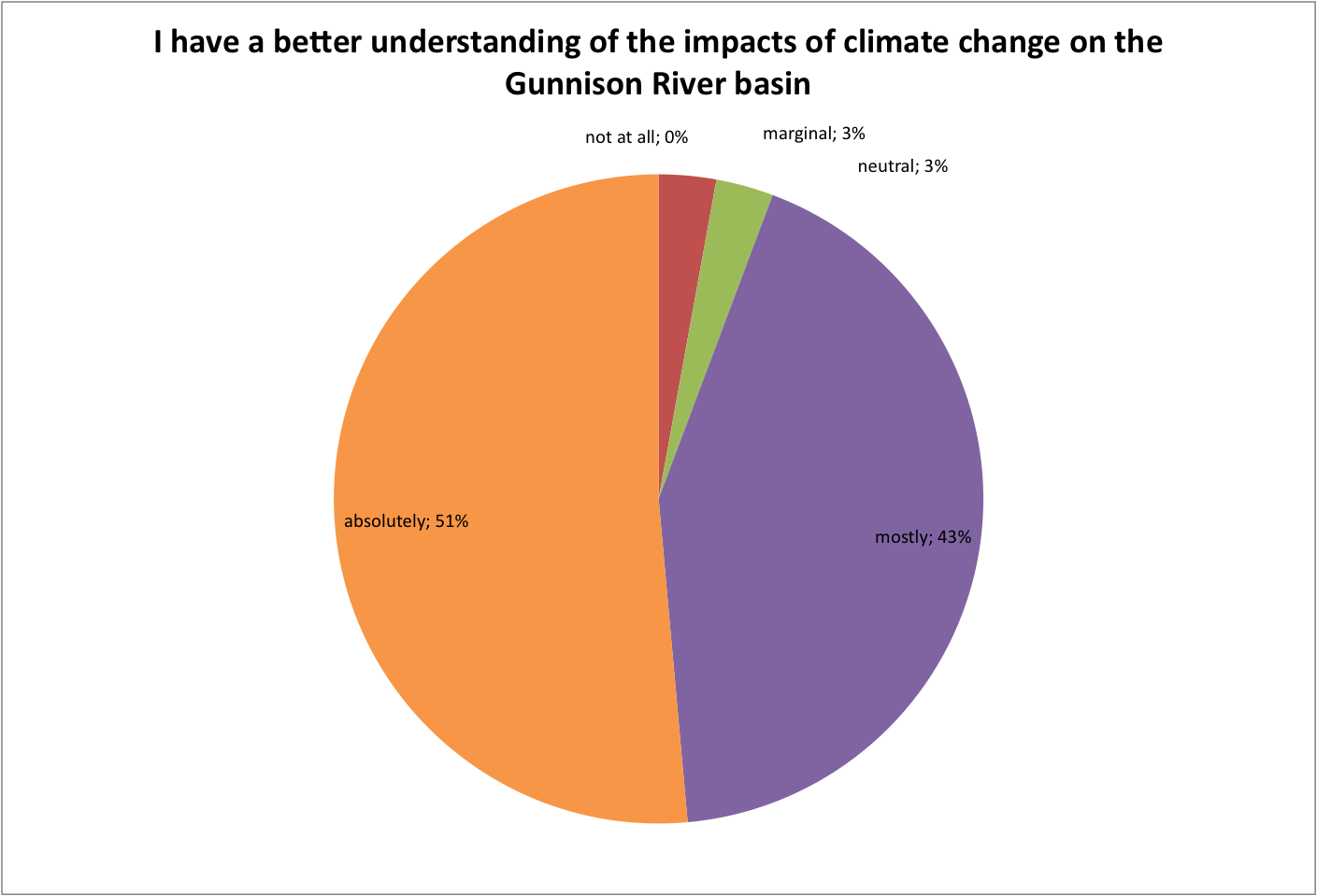


Figure S8. Gunnison River basin workshop participants’ opinions about how the workshop affected their understanding of climate change effects on the Gunnison River basin (N = 35).


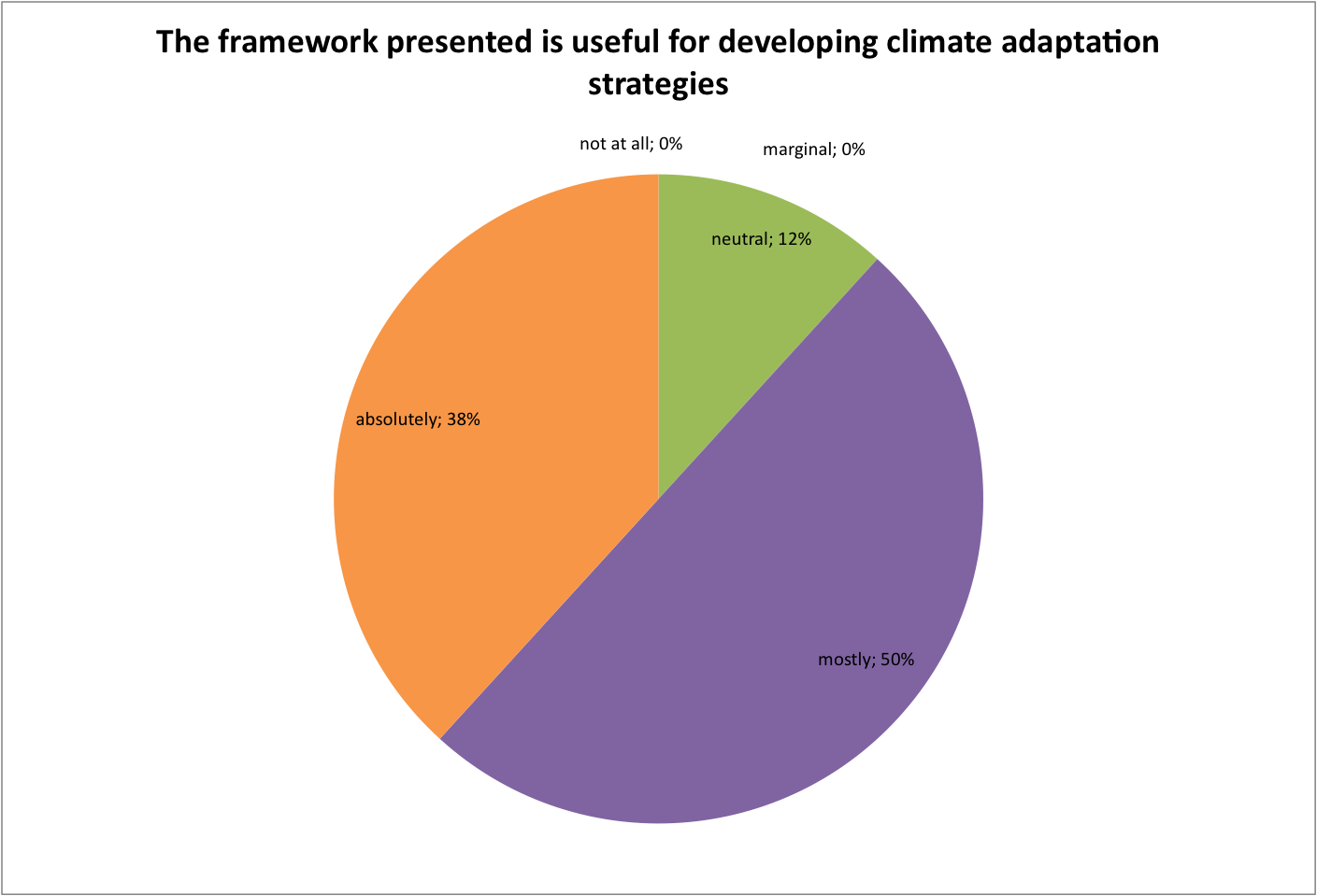


Figure S9. Gunnison River basin workshop participants’ opinions about the usefulness of the Adaptation for Conservation Targets (ACT) planning framework (N = 34).


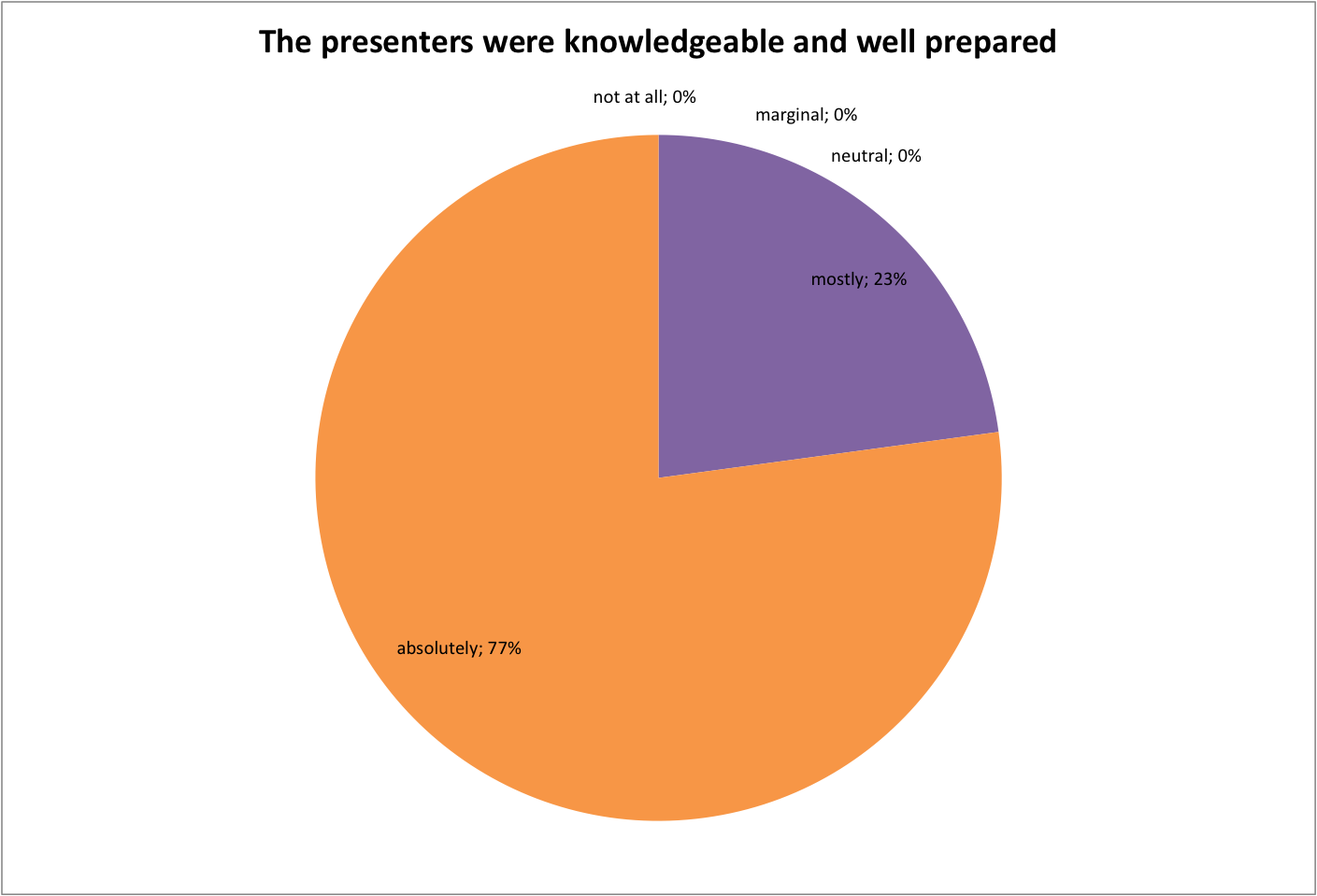


Figure S10. Gunnison River basin workshop participants’ opinions about presenters (N = 35).


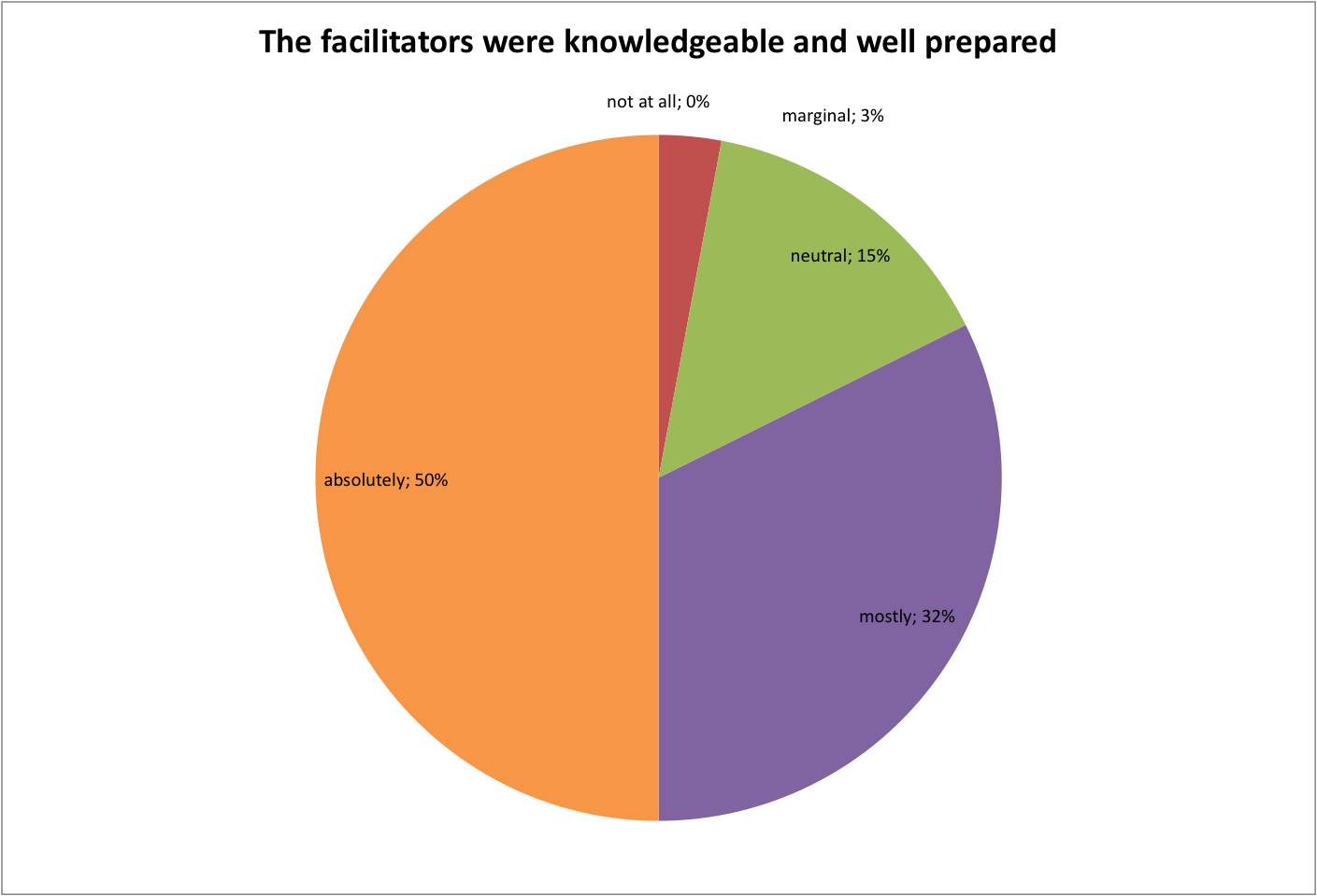


Figure S11. Gunnison River basin workshop participants’ opinions about the workshop facilitators (N = 34).


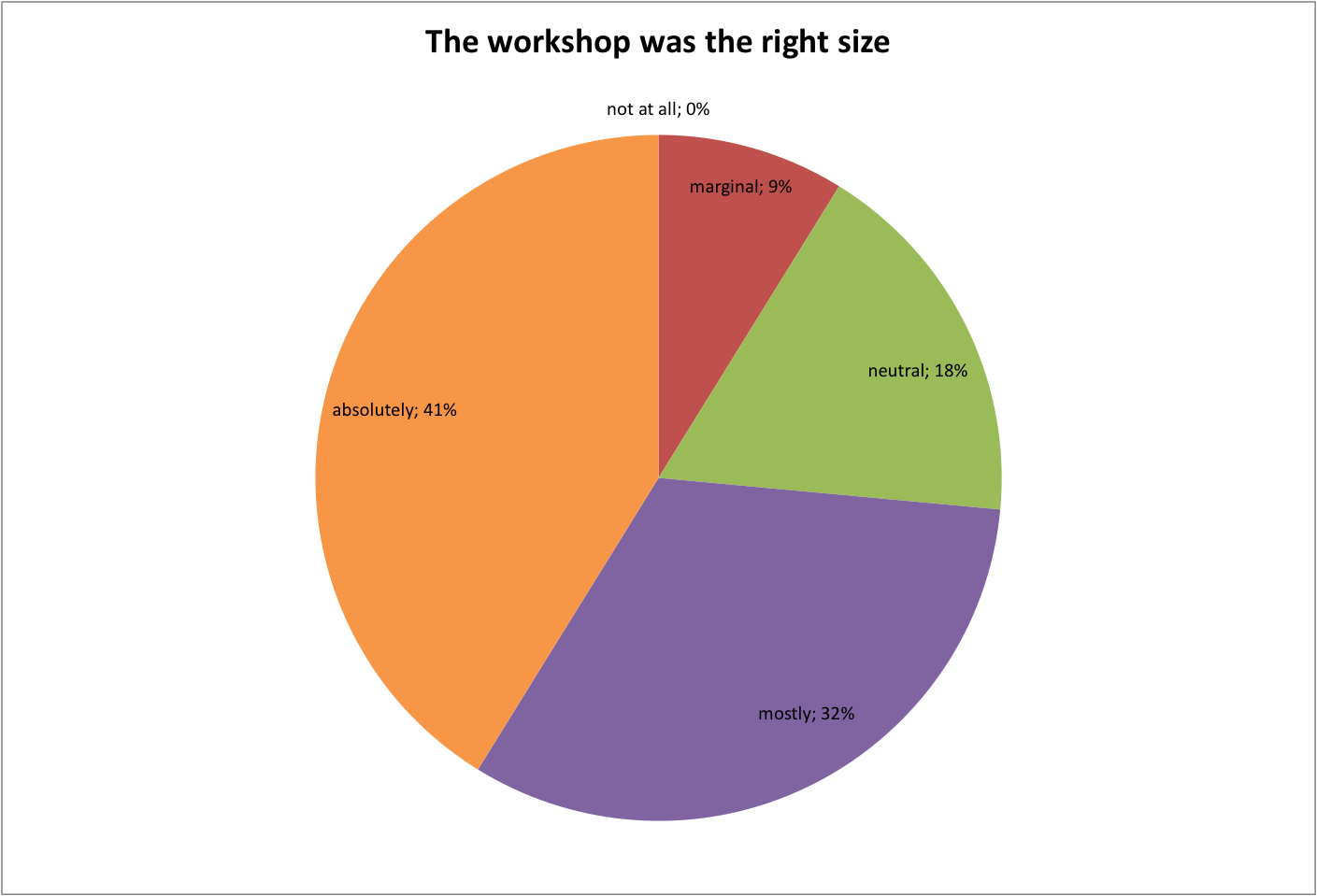


Figure S12. Gunnison River basin workshop participants’ opinions about the size of the workshops (N = 34).


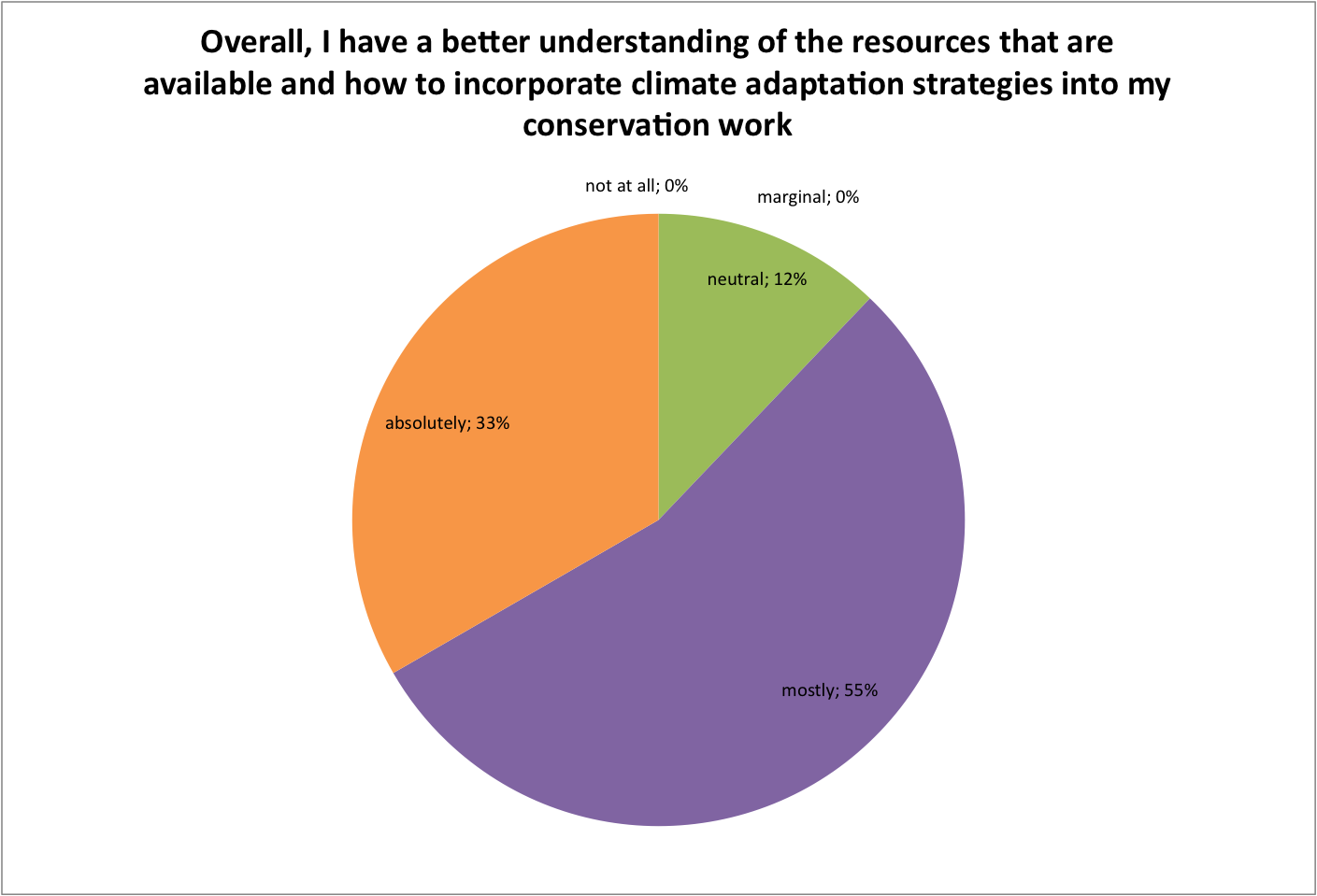


Figure S13. Gunnison River basin workshop participants’ opinions about the overall usefulness of the workshop (N = 33).


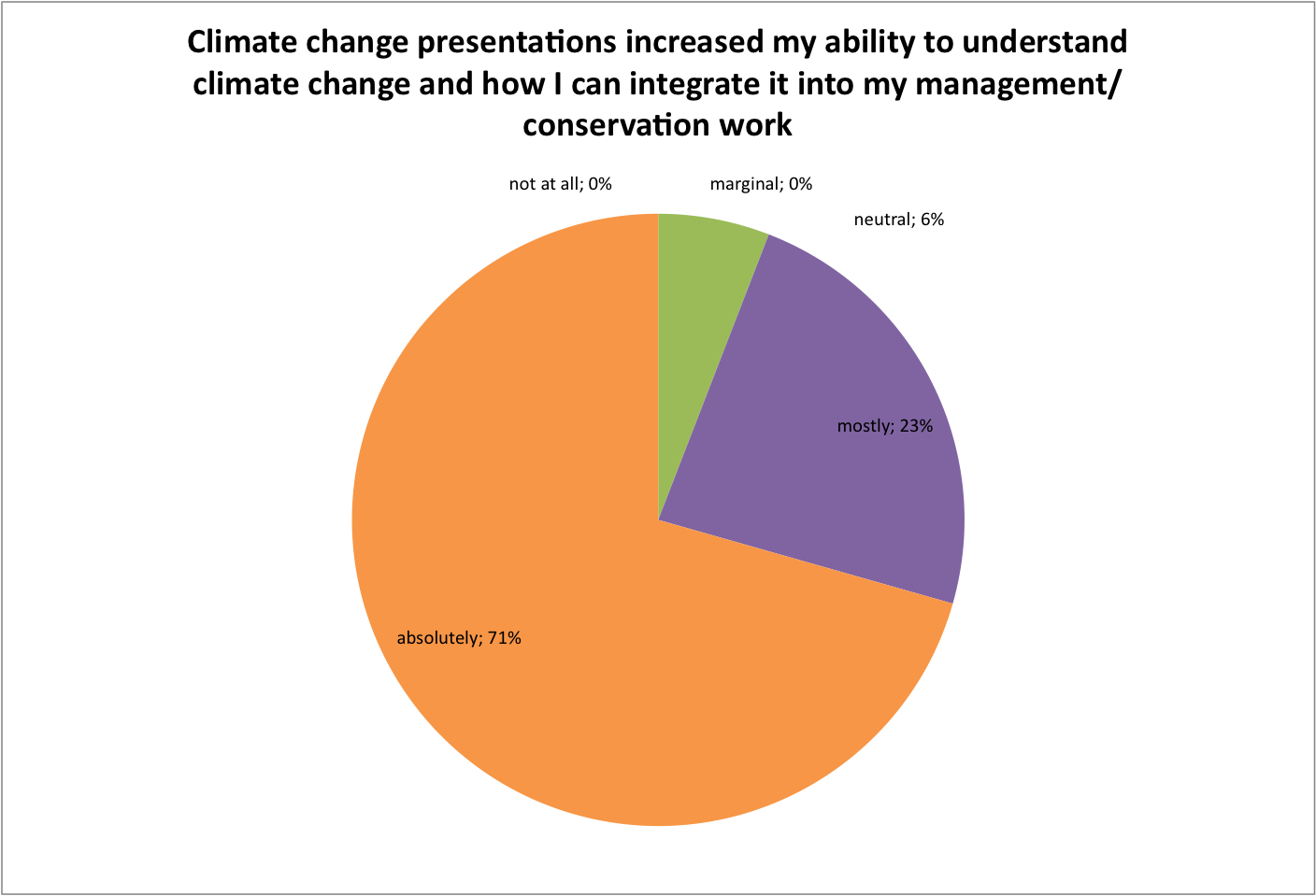


Figure S14. Four Forest Restoration Initiative workshop participants’ opinions about the introductory climate change presentations (N = 17).


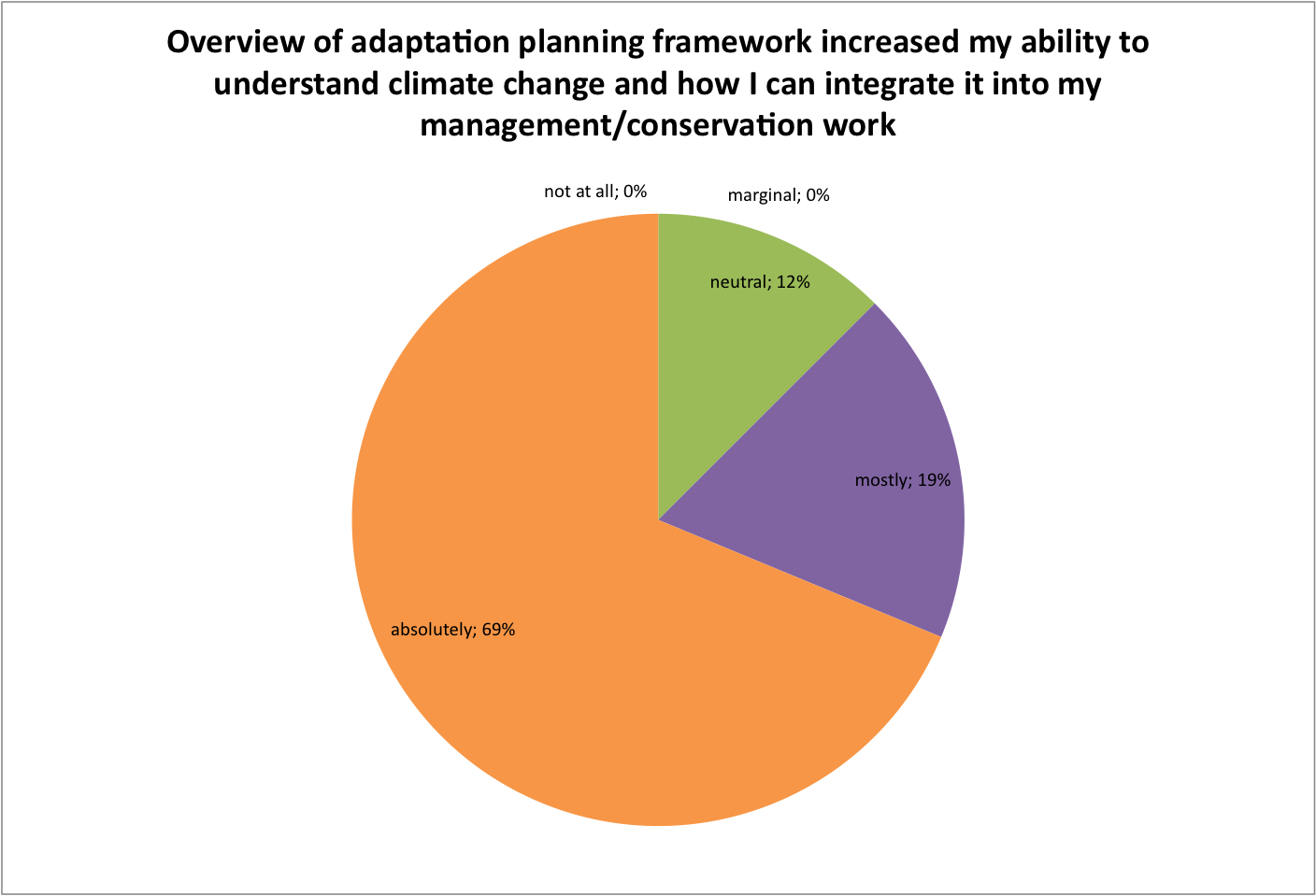


Figure S15. Four Forest Restoration Initiative workshop participants’ opinions about the introductory presentation on the Adaptation for Conservation Targets (ACT) planning framework (N = 16).


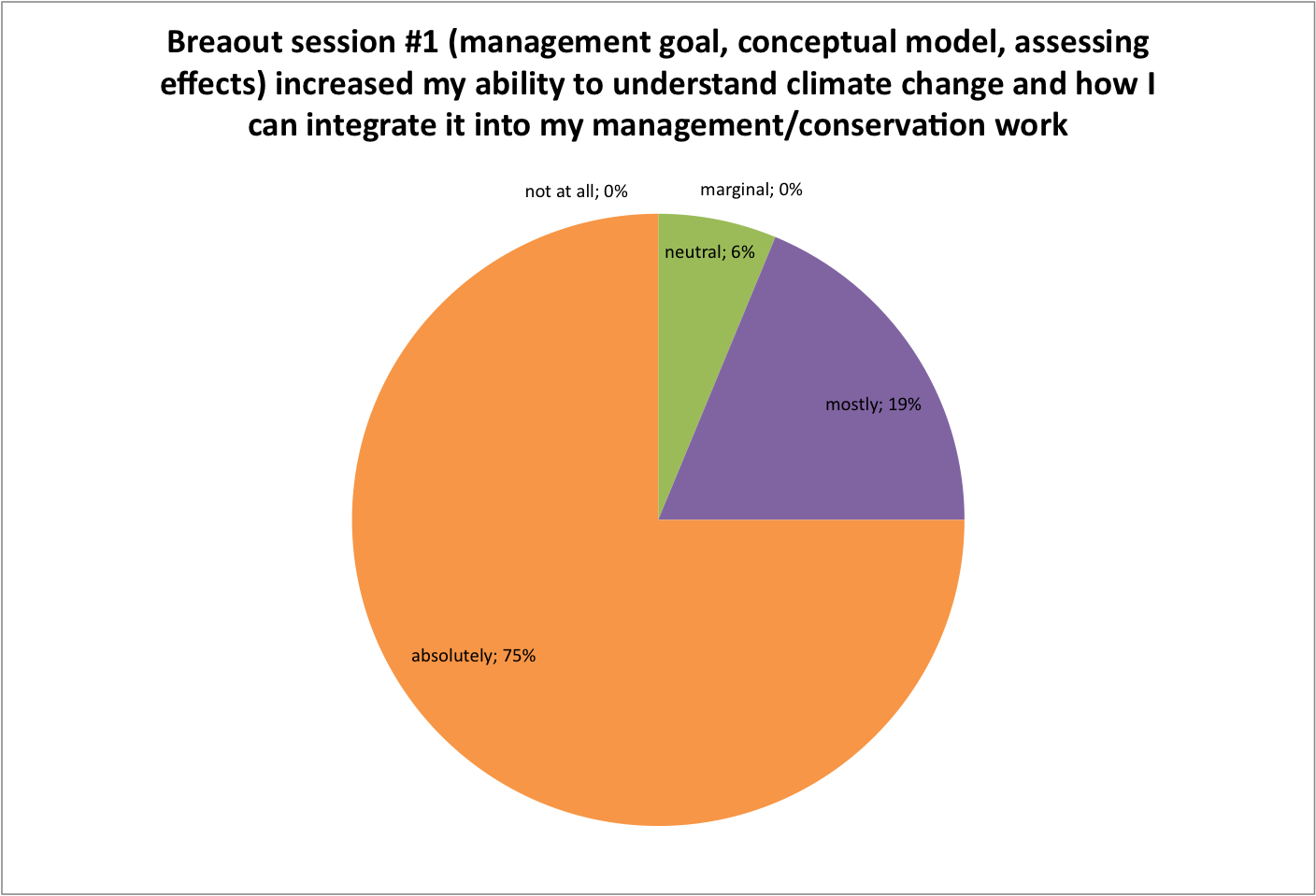


Figure S16. Four Forest Restoration Initiative workshop participants’ opinions about the first breakout session when small groups specified management goals, refined conceptual models, and assessed climate change effects for selected species, ecological processes or ecosystems (N = 16).


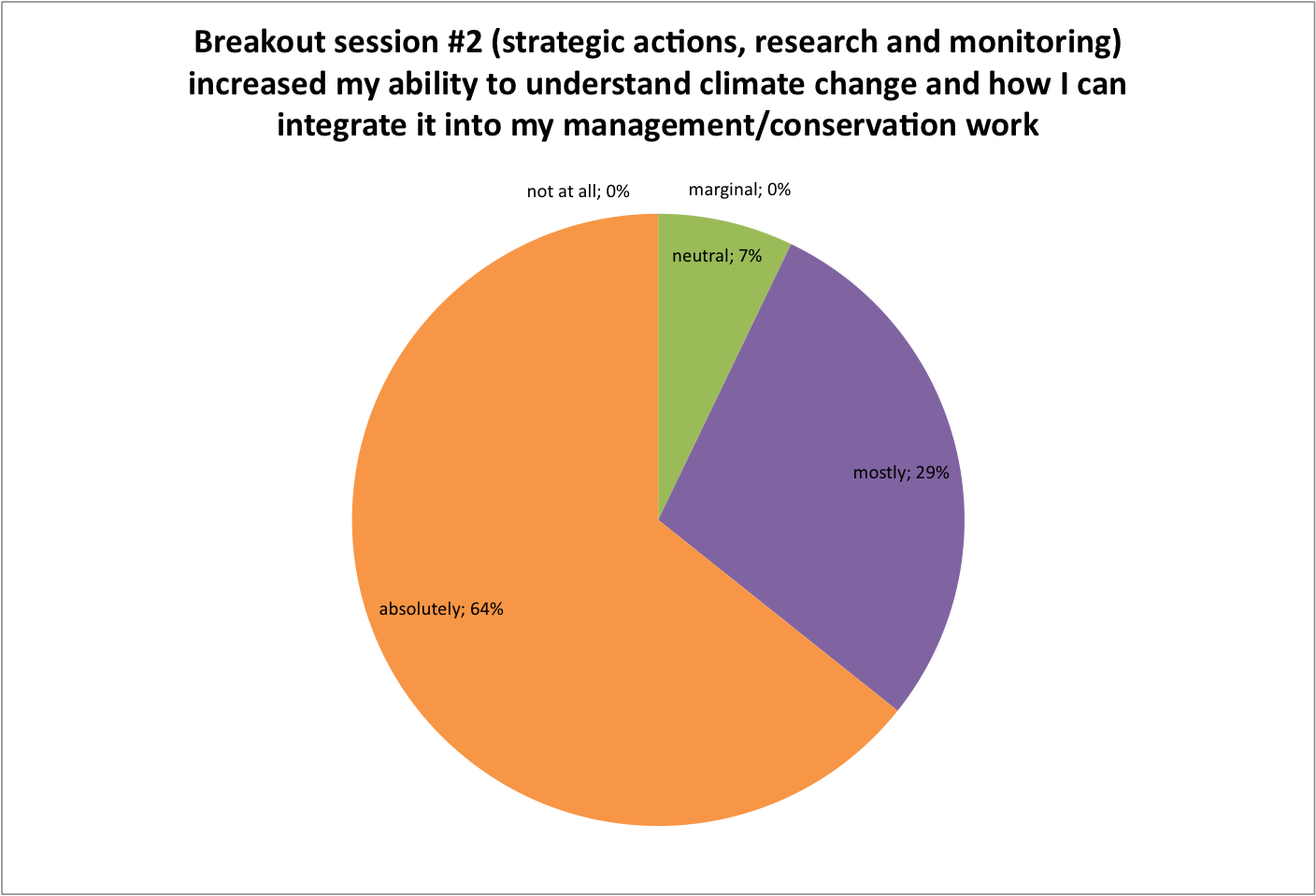
Figure S17. Four Forest Restoration Initiative workshop participants’ opinions about the second breakout session when small groups identified strategic adaptation actions, revisited management goals, and identified research and monitoring priorities (N = 14).


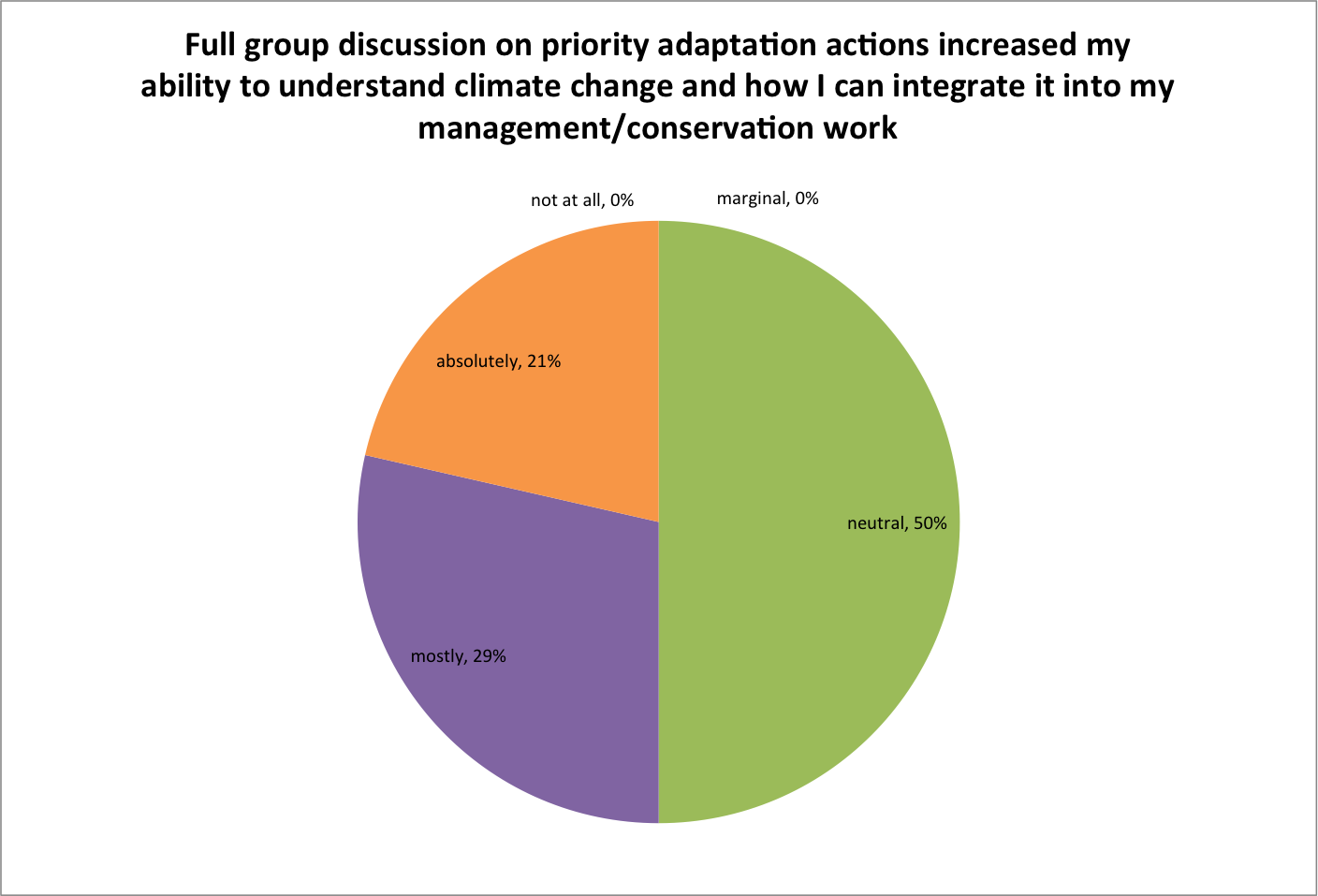
Figure S18. Four Forest Restoration Initiative workshop participants’ opinions about the full-group discussions about priority strategic adaptation actions from each breakout session (N = 14).


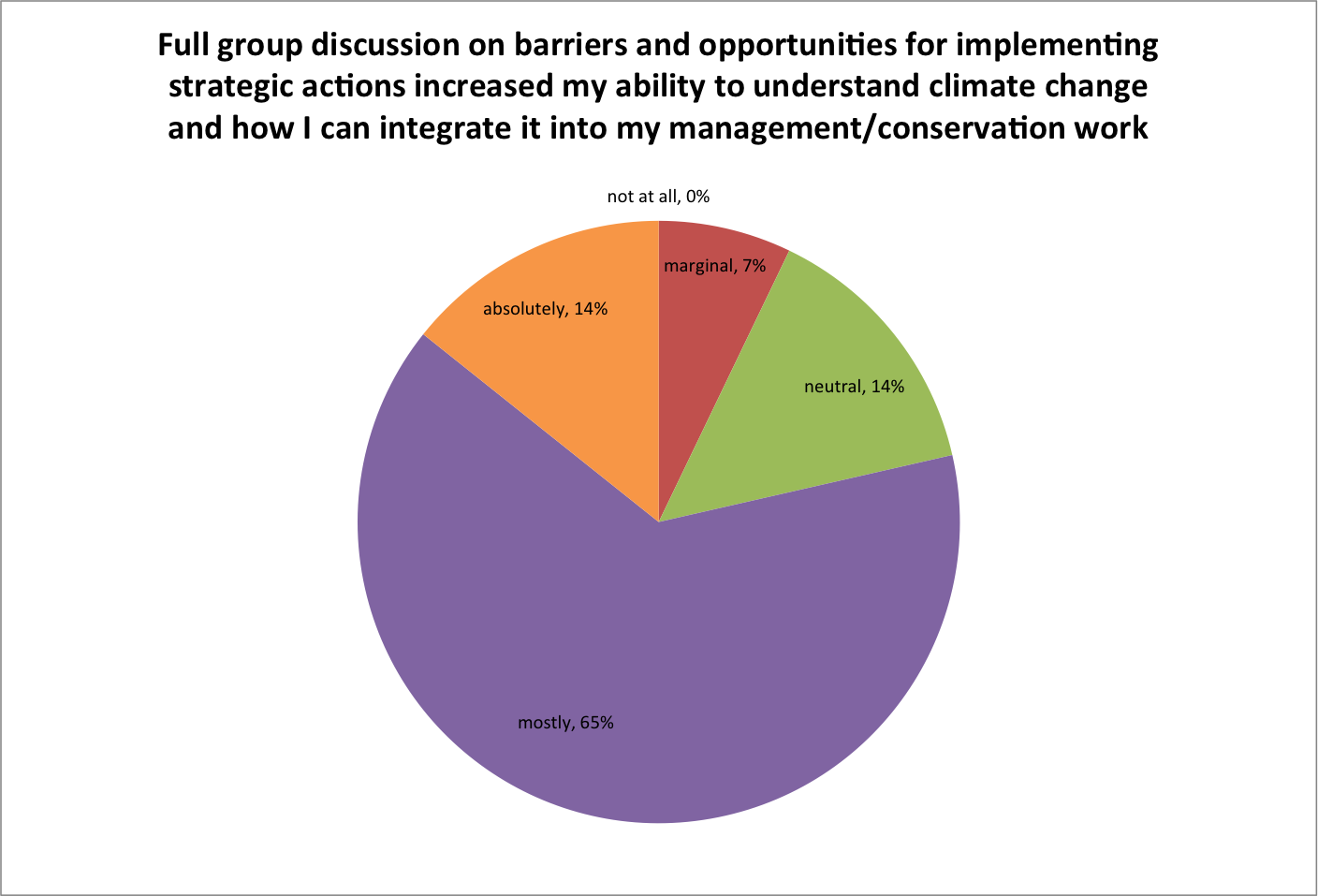


Figure S19. Four Forest Restoration Initiative workshop participants’ opinions about the full-group discussion on barriers and opportunities for implementing strategic adaptation actions (N = 14).


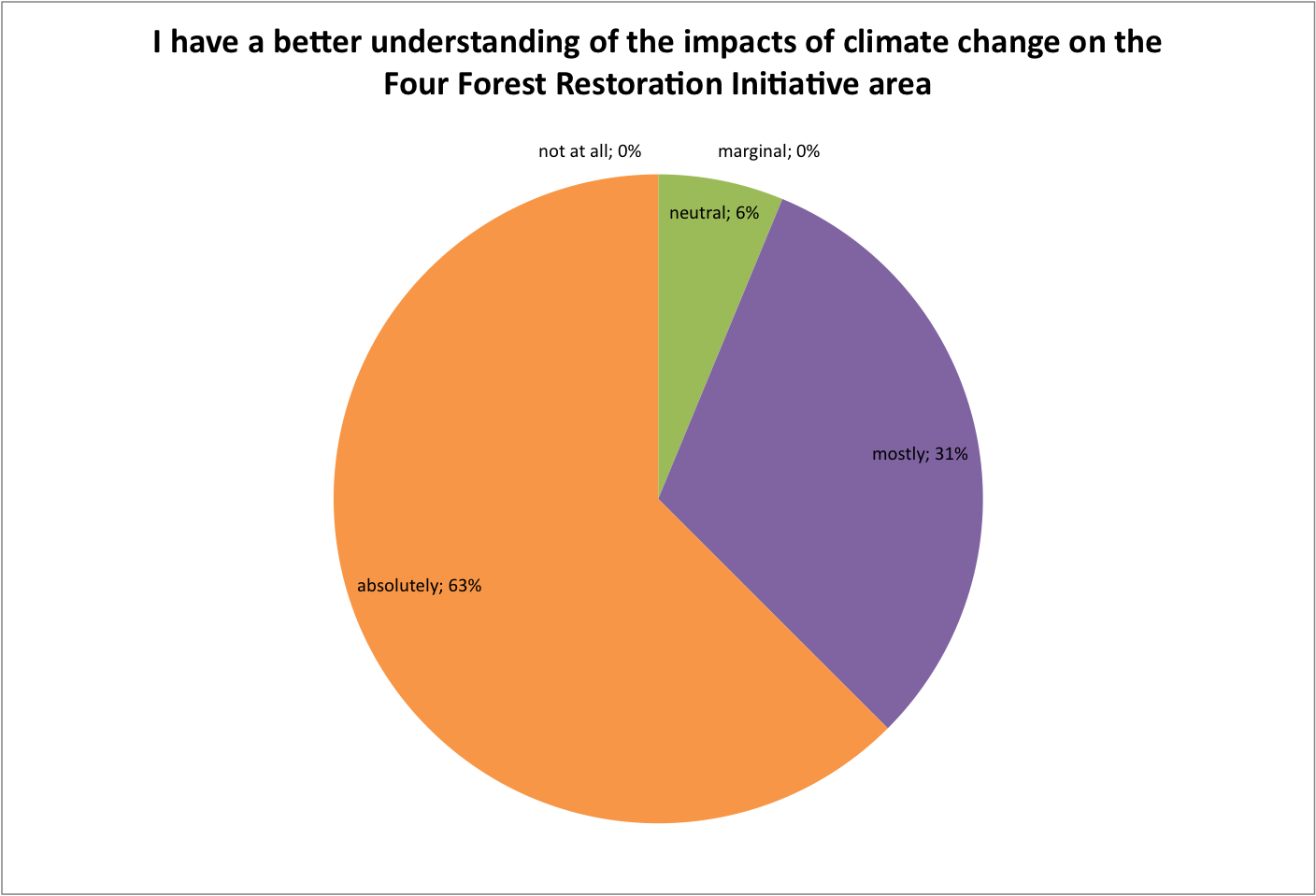


Figure S20. Four Forest Restoration Initiative workshop participants’ opinions about how the workshop affected their understanding of climate change effects on the Four Forest Restoration Initiative area (N = 16).


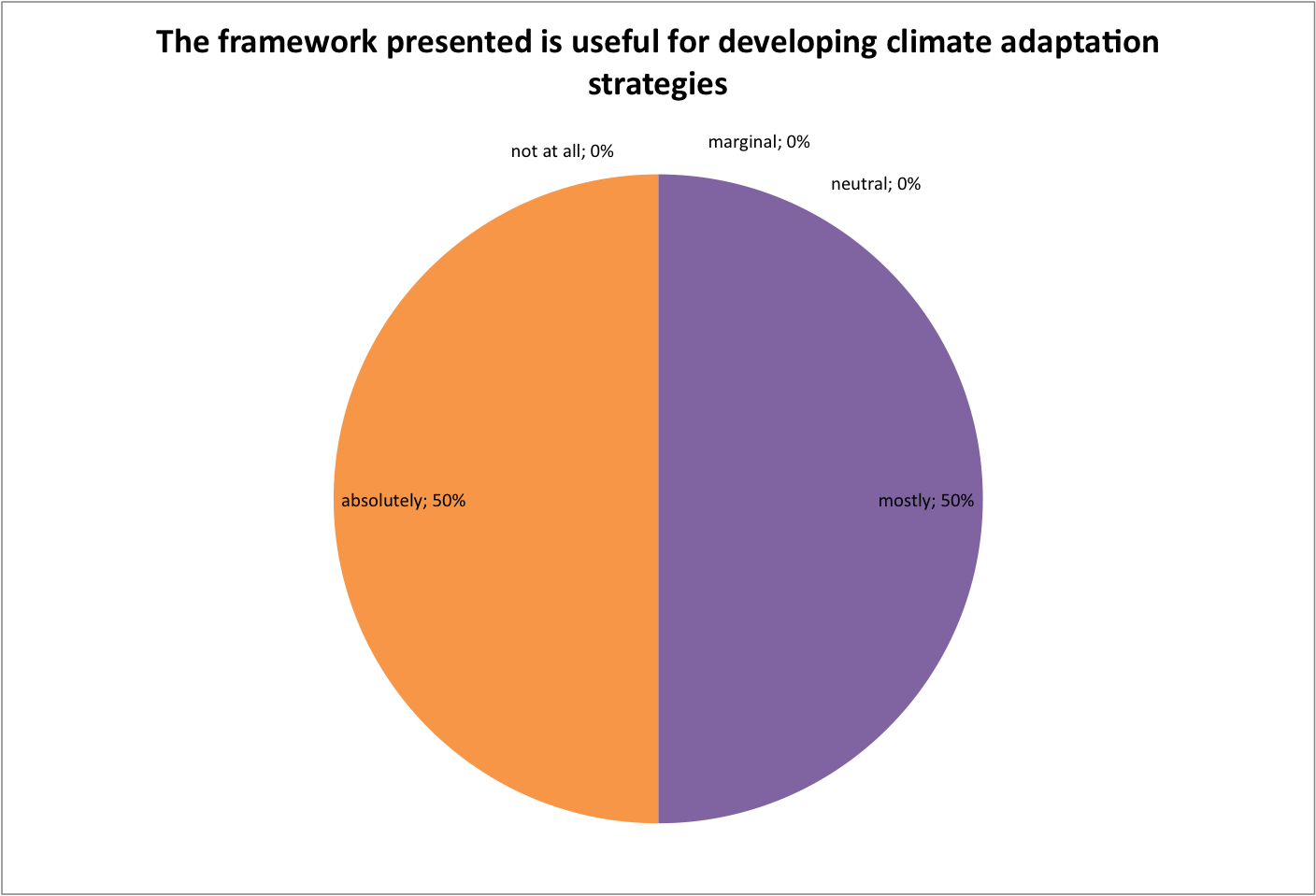


Figure S21. Four Forest Restoration Initiative workshop participants’ opinions about the usefulness of the Adaptation for Conservation Targets (ACT) planning framework (N = 16).


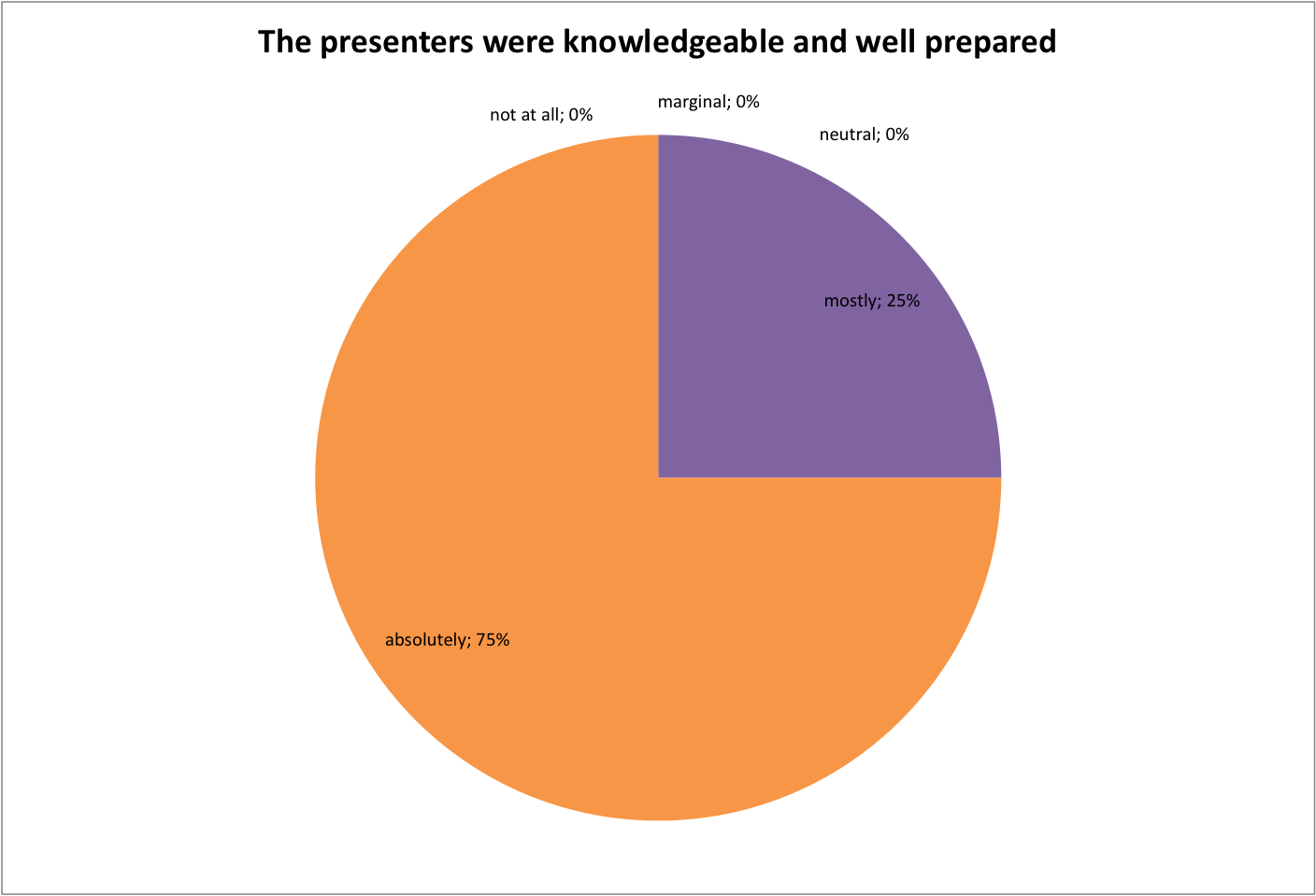


Figure S22. Four Forest Restoration Initiative workshop participants’ opinions about presenters (N = 16).


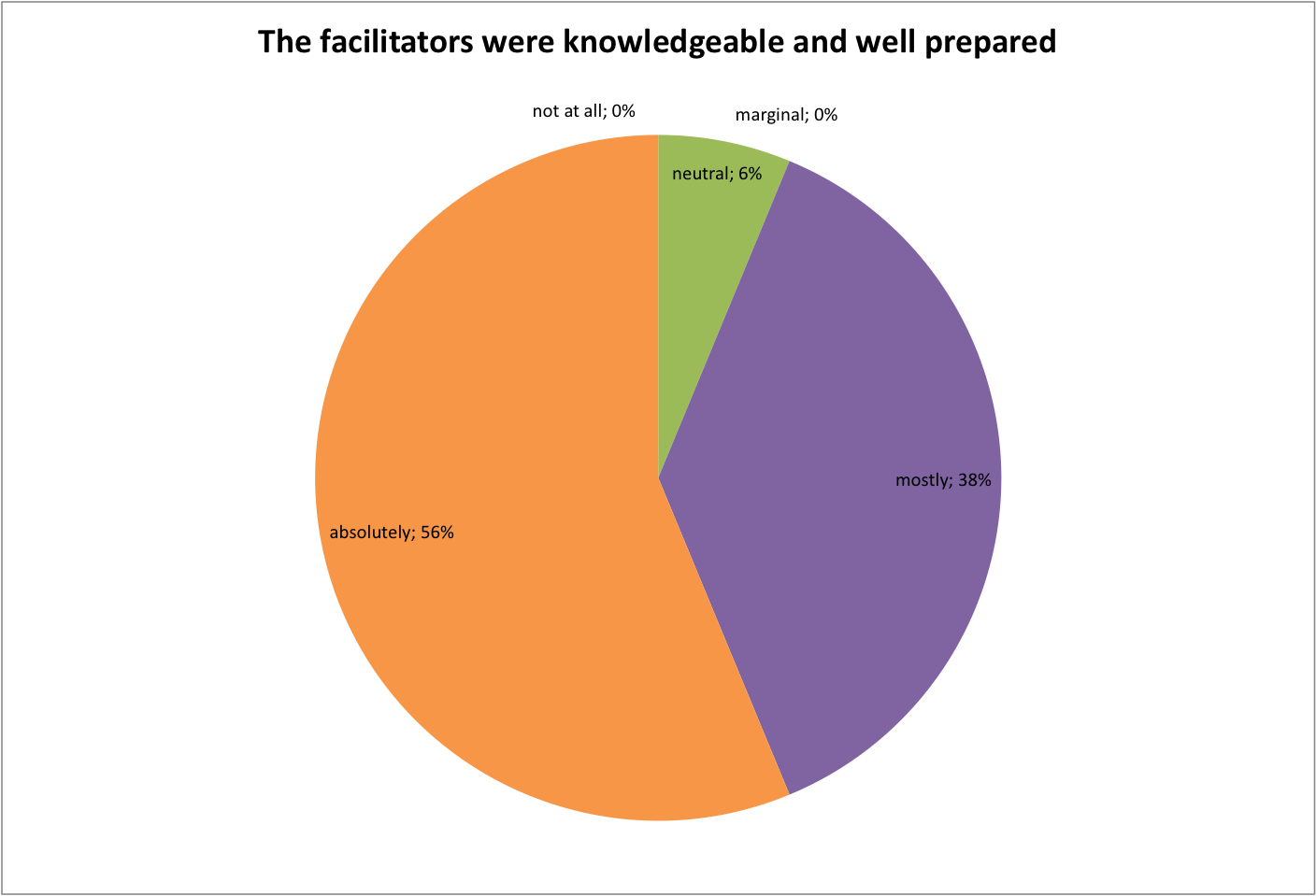


Figure S23. Four Forest Restoration Initiative workshop participants’ opinions about the workshop facilitators (N = 16).


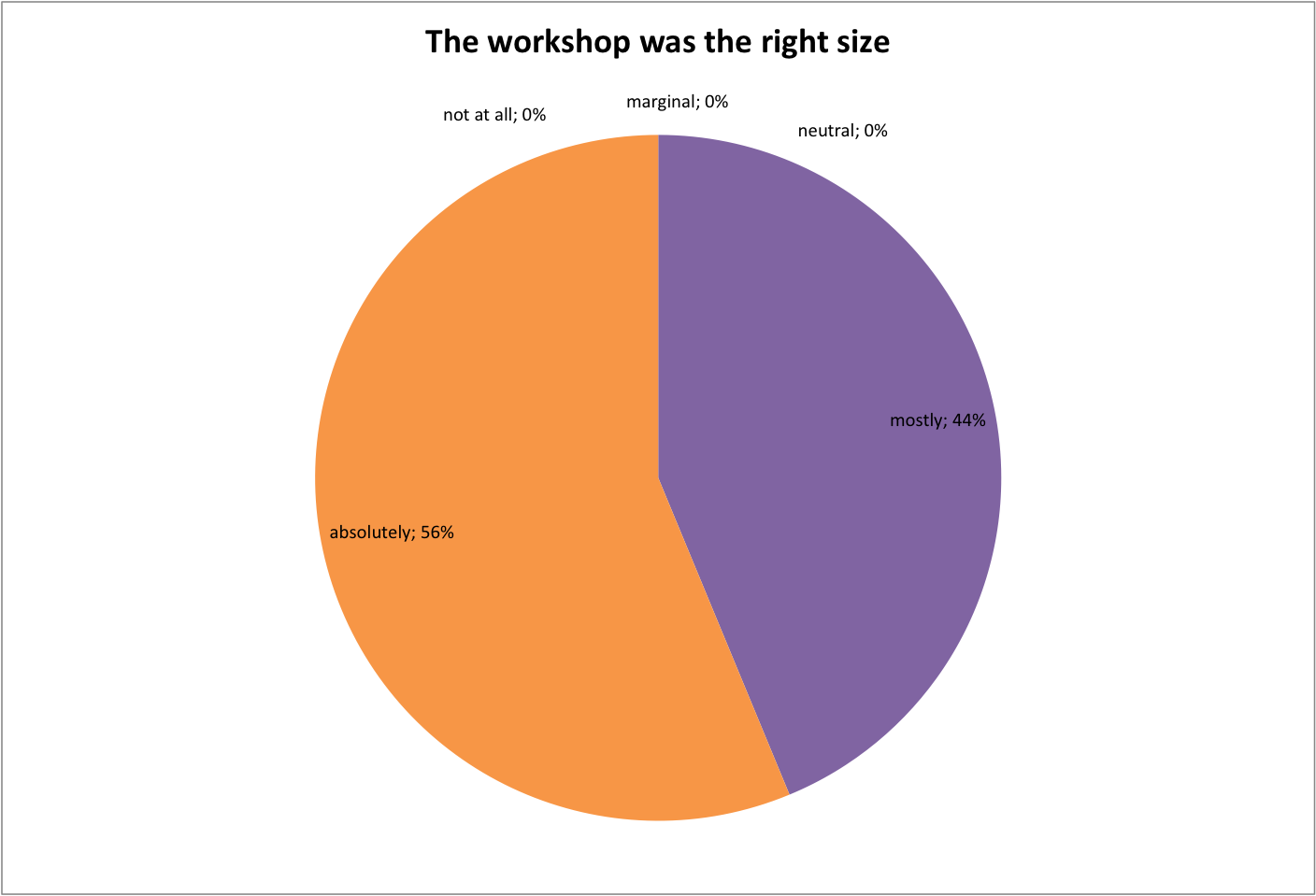


Figure S24. Four Forest Restoration Initiative workshop participants’ opinions about the size of the workshops (N = 16).


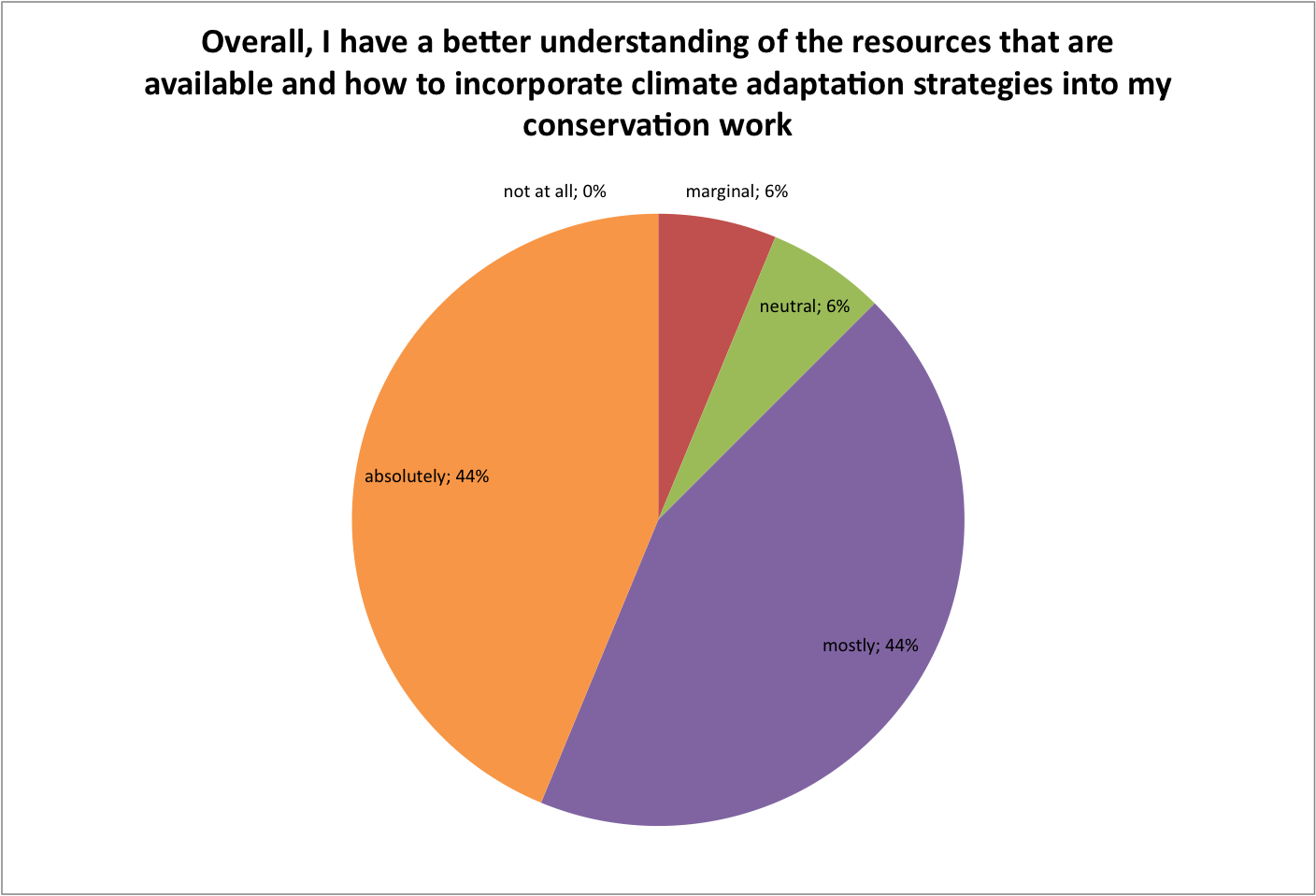


Figure S25. Four Forest Restoration Initiative workshop participants’ opinions about the overall usefulness of the workshop (N = 16).
